# Repurposing landscape of N-acetylcysteine: A scoping review of reviews toward clinical translation

**DOI:** 10.1371/journal.pone.0360434

**Published:** 2026-10-09

**Authors:** Mohammed Khaled Al Alaili

**Affiliations:** Independent Researcher, Clinical Pharmacy, Saida, Lebanon; Michigan Medicine, UNITED STATES OF AMERICA

## Abstract

**Background:**

Growing interest in drug repurposing and oxidative stress has heightened attention on N‑acetylcysteine (NAC). Despite the abundance of reviews across diverse clinical domains, clinical translation remains limited. This review seeks to map the existing evidence, identify critical gaps, and outline priorities for future research.

**Methods:**

Five databases were systematically searched to identify relevant reviews based on predetermined inclusion and exclusion criteria. Comprehensive data extraction enabled meaningful evidence mapping. To enhance rigor, critical appraisal was conducted using two validated tools assessing methodological quality and risk of bias, thereby allowing stratification of evidence. Additionally, the corrected covered area (CCA) was calculated to detect potential evidence inflation or redundancy.

**Results:**

Among the 140 retrieved reviews spanning 17 clinical domains, only 63 were systematic reviews with meta‑analysis, representing the highest level of evidence included in this study. Approximately half of these supported NAC repurposing. Beyond its antioxidant, anti‑inflammatory, and glutamatergic modulatory roles, which were most frequently reported, several additional mechanisms have been proposed. The number of NAC reviews has increased over time, with most originating from China and the United States. Appraisals revealed a high prevalence of critically low‑quality systematic reviews, alongside a notable proportion of reviews with low risk of bias. Finally, CCA analysis demonstrated substantial overlap and redundancy across domains, particularly within subdomains.

**Conclusion:**

This novel overview mapped the extensive NAC evidence landscape, highlighting its pleiotropic roles and repurposing potential while revealing possible research waste and critical gaps across domains and evidence levels. Psychiatry, reproductive medicine, nephrology, pulmonology, and toxicology emerged as priority clinical domains for future exploration. However, the overall inconsistency in review certainty remains a barrier to the integration of NAC into clinical guidelines. By delineating research priorities and highlighting new avenues, this review underscores the need for precision medicine approaches to guide future NAC clinical implementation and research.

## 1. Introduction

N-acetylcysteine (NAC) is a long-recognized molecule with antioxidant properties that possesses exceptional mechanistic versatility [1,2]. Its sulfur content enables glutathione synthesis, forming the basis of its adoption in paracetamol toxicity, where it mitigates N-acetyl-p-benzoquinone imine (NAPQI) accumulation [3]. Although less potent than cysteine and glutathione, NAC’s free sulfur group allows it to disrupt disulfide bonds in mucin proteins, supporting its use as a mucolytic adjuvant in pulmonary conditions such as acute and chronic bronchitis, pneumonia, cystic fibrosis and diagnostic procedures [4]. These remain the only FDA‑approved and guideline‑endorsed clinical indications for NAC [5]. Nevertheless, growing interest in oxidative stress [6,7] and adjunctive therapies [8] has expanded NAC’s off‑label applications, such as in non‑paracetamol liver and kidney injury. Its broad biological plausibility and network pharmacology have advanced more rapidly than definitive evidence synthesis [9]. Its mechanistic diversity has rationalized its wide applicability potential, as NAC has been proposed to act as an antioxidant precursor, direct antioxidant, glutathione modulator, redox regulator, and mitochondrial stabilizer [2]. Meta‑analytic evidence further demonstrates its significant impact on oxidative stress and inflammatory biomarkers [10].

Drug repurposing, also termed repositioning, refers to the use of a drug beyond its conventional or approved indications [11]. This approach has accelerated in recent years and is increasingly advocated to reduce polypharmacy and promote rational drug use, particularly for low‑cost and globally accessible therapeutics [12]. Outcomes of repurposing may encompass clinical and comparative effectiveness, tolerability, mechanistic or biomarker insights, and population‑specific benefits [13]. NAC repurposing has emerged through recognition of shared pathobiological mechanisms across heterogeneous clinical conditions [14]. The expanding interest in NAC reflects a broader shift toward multi‑target agents capable of modulating convergent biological pathways implicated in both acute and chronic diseases [15]. While many off‑label indications are extrapolated from its antioxidant and anti‑inflammatory properties, additional mechanisms, such as glutamatergic modulation, have shown promise in psychiatric and neurological disorders [1]. Collectively, these features underscore NAC as a multi-system molecule with strong potential candidacy for repurposing, supported by its favorable safety profile, affordability, and wide availability, particularly in resource‑limited settings [16]. For instance, in low-income countries, where non-acetaminophen acute liver injury is more prevalent [17], NAC would stand to offer greater benefit from repurposing, in contrast to high-income countries where its primary use remains as an antidote for acetaminophen-induced liver failure. However, no single indication predominates, raising critical questions about why NAC demonstrates variable success and inconsistent clinical efficacy despite compelling mechanistic rationale. This inconsistency represents the central challenge addressed by this review.

Over the years, numerous studies have investigated NAC, prompting an exponential rise in reviews aimed at consolidating evidence and identifying new indications [18–21]. However, the absence of umbrella‑level synthesis focusing on NAC raises some concerns. Previous reviews have largely focused on single indications, providing valuable but isolated insights. Despite extensive exploration, no new indications have been firmly established, and the available literature remains limited, with evidence dispersed across specialties and characterized by heterogeneity. This redundancy and uncertainty—where signals vary in strength—contributes to clinician confusion and guideline development delay. Signal dilution further obscures promising indications within fragmented evidence. To date, the clinical field lacks a unified, mature translational framework across indications. In particular, there is limited understanding of the comparative maturity of evidence, areas of saturation versus scarcity, and the degree of concordance between mechanistic rationale and clinical translation.

In light of these challenges, this scoping review of reviews was undertaken to address existing gaps and, to our knowledge, provide the first comprehensive cross‑disciplinary synthesis, unlike umbrella reviews which aim to synthesize effect sizes. Its objectives are to map and characterize the breadth of evidence on NAC repurposing across clinical indications and domains, and to identify therapeutic signals and mechanistic themes. Furthermore, it seeks to uncover patterns of evidence convergence and translational discordance, while highlighting knowledge gaps, methodological trends, and future priorities for researchers and policymakers. Specifically, this review aims to clarify where trials should be prioritized and where NAC demonstrates clear promise, thereby informing clinical practice and precision repurposing strategies.

## 2. Methods

### 2.1. Protocol and reporting standards

This review was conducted in accordance with the Joanna Briggs Institute (JBI) framework [22]. Reporting adhered to the Preferred Reporting Items for Systematic reviews and Meta-Analyses extension for Scoping Reviews (PRISMA-ScR) guidelines [23], with the corresponding checklist provided in S1 File. The review protocol was prospectively registered on the Open Science Framework (OSF), ensuring transparency and reproducibility, and is accessible via the following link: https://doi.org/10.17605/OSF.IO/3VE5J. No major deviations occurred. Ethical approval was not required for this study because it is a review of previously published, publicly available literature. No human participants, animals, tissues or identifiable data were involved.

### 2.2. Search strategy and selection criteria

A pilot search was conducted in November 2025, followed by a comprehensive search initiated in December 2025 after protocol registration and completed in February 2026. To ensure consistency and reduce single-author bias, a subset of the search was replicated to verify results. The overall review process is illustrated in the flow chart in Fig 1. Five databases—TRIP (Turning Research Into Practice), CDSR (Cochrane Database of Systematic Reviews), Google Scholar, Epistemonikos, and PubMed—were systematically searched using relevant keywords and filters, targeting publications from the past ten years. The search strategy was intentionally based on keywords to maximize search sensitivity and ensure broad coverage, consistent with the objectives of a scoping review. The same conceptual search strategy was adapted for each database while accounting for differences in database-specific syntax and search functionality. Where database features (e.g., study design filters) were unavailable, equivalent restrictions were applied during the screening process through the predefined eligibility criteria. A targeted operator “allintitle:” was employed in Google Scholar to minimize noise and heterogeneity, ensuring that retrieved reviews primarily addressed NAC as the main intervention of interest. This was particularly relevant given our search string revolved mainly around NAC, and the operator ensured that records captured NAC as a central focus rather than incidental mention. The approach aligns with the review question and predefined eligibility criteria, thereby enhancing the specificity of our search strategy. All available results (n = 67) in Google Scholar were screened manually. A ten-year window was selected to balance comprehensiveness with feasibility and because older primary studies are already incorporated within existing reviews. This ensured focus on the most recent and clinically relevant syntheses without unnecessary duplication. All types of reviews were included. Only English-language studies were included to enhance international applicability and reporting consistency. Detailed search parameters (PCC framework, filters, and terms), along with inclusion and exclusion criteria, are presented in Fig 2. Since no specific search field was restricted, all vocabulary terms related to the keyword *N-acetylcysteine* were considered in PubMed. For consistency across databases, *N-acetylcysteine* was the sole search term selected. The search strategy developed for the main database (PubMed) is presented as an example of the search approach. This reporting approach aligns with PRISMA-ScR guidance, which requires at least one detailed database strategy. To enhance rigor and transparency, the characteristics of excluded articles (abstract-level), including the number, type, and topics of non-NAC-focused and inaccessible studies, were mapped and are presented in Fig 3. Indirect references to NAC were excluded to ensure the review remained treatment-focused rather than disease-focused, preventing data inflation and minimizing heterogeneity. This approach also reduced noise, ensuring alignment with JBI and PRISMA principles, which emphasize mapping evidence directly relevant to the review question. Duplicate records were removed manually, reference lists were screened for additional eligible studies, and titles and abstracts were systematically screened.

**Fig 1:**
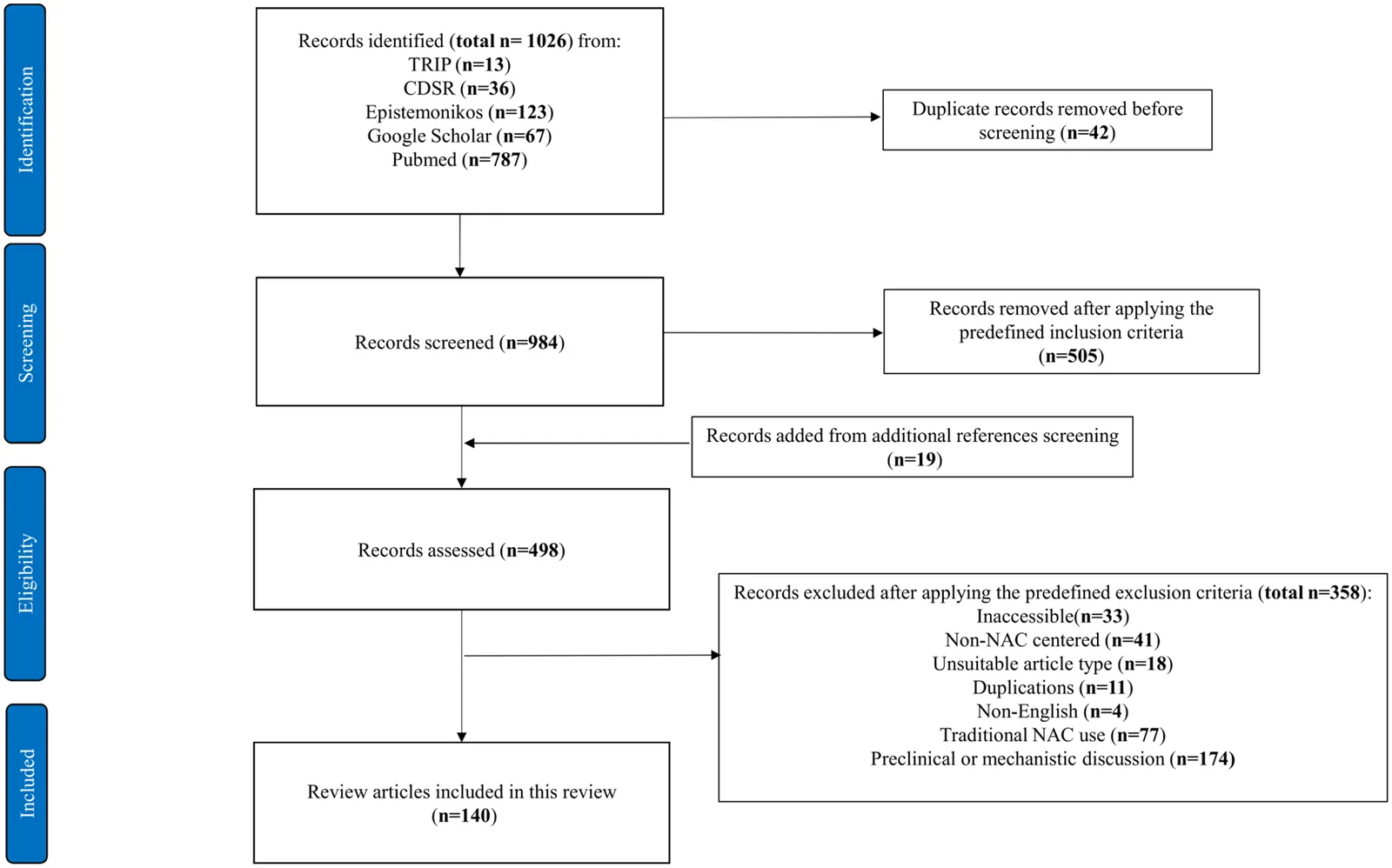
PRISMA flowchart. The chart shows the number of records identified, screened, excluded, and retained, with reasons for exclusion. This ensures reproducibility and transparency in study selection. CDSR: Cochrane Database of Systematic Reviews. NAC: N-acetylcysteine. TRIP: Turning Research Into Practice. PRISMA: Preferred Reporting Items for Systematic reviews and Meta-Analyses.

**Fig 2:**
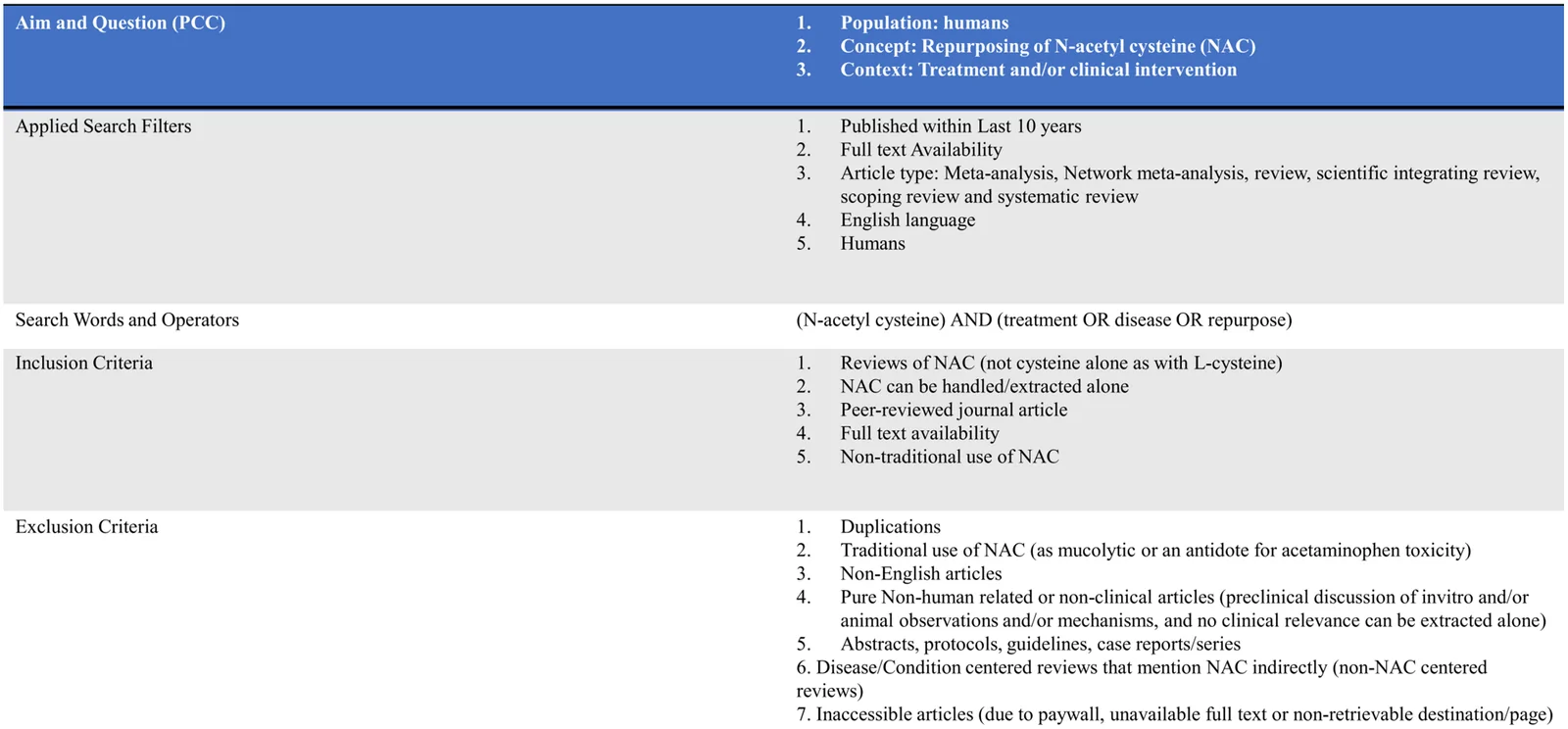
Search strategy. This figure outlines the review concept and framework, the search filters/terms and the adopted inclusion/exclusion criteria. This strategy aligns with the reporting guidelines.

**Fig 3:**
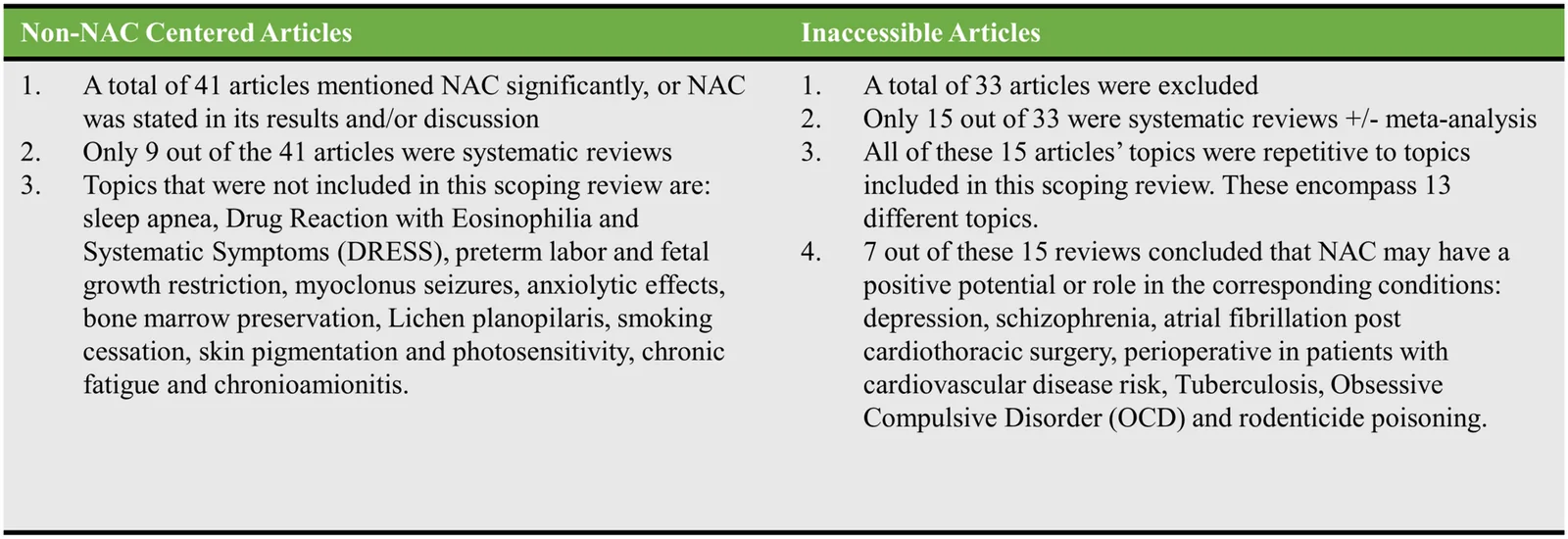
Summary of excluded studies. This chart displays the characteristics of inaccessible and indirect excluded reviews. This mapping demonstrates transparency in the screening process.

### 2.3. Data extraction

To minimize bias and enhance transparency, data extraction was performed using a predetermined extraction sheet. This sheet captured the following variables: title, author, publication year, country/review origin, clinical indication, clinical domains derived through inductive thematic reasoning and analysis of the retained literature, review type, number and type of included studies, population characteristics, conclusions, signal detection categorized as supportive, neutral, or against NAC use based on each article’s conclusion without subjective reinterpretation, limitations, evidence certainty (if reported), dose ranges, route of administration, and mechanism of action. Any nuances such as mixed outcomes, biomarker-only effects, subgroup findings, safety concerns, and multiple indications were documented, if available, in the ‘limitations’ column of the main extraction table in the supplementary file. To ensure the clarity, consistency and accuracy of the extraction process, a small random subset of five included studies was checked by an independent research colleague. Discrepancies were minimal and resolved via discussion. This step served as an informal verification and quality-control measure rather than formal dual screening; therefore, agreement statistics were not calculated. The complete extraction table is provided in S2 File.

### 2.4. Critical appraisal

Although critical appraisal is optional in scoping reviews, it was applied as a descriptive component to ensure rigor, credibility and to enhance interpretability. Only systematic reviews with or without meta-analyses were eligible, with no exclusions applied, aligning with the scoping review objective of mapping and contextualizing the evidence rather than excluding studies based on quality. Review quality was assessed using AMSTAR-2 (A MeaSurement Tool to Assess systematic Reviews) [24], which categorizes studies as critically low, low, moderate, or high. Seven critical domains—protocol registration, adequacy of the literature search, justification of exclusions, risk of bias assessment, appropriateness of meta-analysis (if conducted), consideration of risk of bias in results, and evaluation of publication bias—were prioritized. If two critical flaws were identified, the review was rated as critically low; one flaw resulted in a low rating. Non-critical flaws were then considered: the presence of two downgraded a high rating to moderate, while the presence of all led to a low rating. Each included systematic review was independently rated. Ratings were then summarized by frequency and domain to enable stratified evidence mapping.

Given the large number of retained studies and the need for stratified, inferential evidence mapping, ROBIS (Risk Of Bias In Systematic reviews) was additionally applied [25]. ROBIS focuses specifically on risk of bias across four domains: study eligibility criteria, identification and selection, data collection and appraisal, and synthesis and findings. If any domain was judged to be of high concern, the overall rating was high risk; if none were of concern, the rating was low risk; and if assessment was not possible, the rating was classified as unclear. To balance feasibility, readability and transparency, only overall individual ratings from both tools, with documented reasons where applicable, were reported and provided in S3 File, while stratified results across domains are visualized in stacked bar charts. Using both tools provides complementary perspectives, methodological rigor and bias assessment, underscoring the robustness of this review’s approach.

### 2.5. Corrected covered area (CCA)

Although overlap analysis is more commonly applied in umbrella reviews, it was calculated in this scoping review to strengthen methodological rigor and add transparency to the evidence mapping and its interpretation. For domains addressing similar conditions or indications with multiple systematic reviews and meta-analyses, given their capacity to generate quantitative certainty and effectiveness, percentage overlap was calculated using the CCA [26]. The purpose was to identify redundancy and mitigate the risk of duplicated primary studies, thereby reducing inflated results and evidence uncertainty, rather than to quantify effectiveness. For example, if three reviews all include the same ten primary studies, the overlap is very high. While this may appear to strengthen confidence in the findings, in reality it inflates the impression of evidence without adding new knowledge, since the same studies are being counted multiple times. The formula applied was: (Nr−Ns)÷(r×Ns−Ns), where Nr represents the total number of included studies across all reviews in the selected domain, Ns denotes the number of unique, non-repeated primary studies, and r indicates the number of reviews within the domain. Results were categorized as 0–5% (slight overlap), 6–10% (moderate), 11–15% (high), and >15% (very high). These categories indicate whether the evidence base is broad and independent or at risk of redundancy and inflated certainty, thereby prompting caution in interpreting the corresponding evidence. To maintain clarity and facilitate reproducibility, full details of matrices for eligible domains, subdomains, and related reviews, along with overlap results, are provided in S3 File. Clinical domains were those derived inductively during data extraction. Subdomains were defined primarily based on shared clinical indications, with overlap estimates used to highlight concentrated clustering within narrower categories.

### 2.6. Data synthesis and software

All calculations, data management, figures, and graphs were conducted and generated using Microsoft Excel (2016). No inferential statistical testing was undertaken. References were organized with Mendeley Reference Manager (v1.19.8).

## 3. Results

### 3.1. Search findings

From the 1,026 articles initially identified across databases, 140 reviews met eligibility criteria and were retained for inclusion in this scoping review, with the full list provided in S4 File. Extracted data are synthesized in Table 1, which presents an evidence-mapping landscape across 17 clinical domains: cardiology [27–30], psychiatry [19, 31–57], pulmonology [20,58–67], oncology [68–70], reproductive medicine [71–81], toxicology [21,82–88], hepatology [89–103], dermatology [104], pain medicine [18,105,106], neurology [47,107–114], nephrology [115–132], sports medicine [133–135], geriatric medicine [136], immunology [137–139], oral medicine [140], endocrinology [141–143], and infectious diseases [144–162]. This breadth highlights NAC’s potential as a multi-system therapeutic candidate.

**Table 1:** Evidence mapping of N-acetylcysteine (NAC) repurposing landscape across clinical domains. The table summarizes clinical indications, signal detection, number of systematic reviews with meta-analysis, study characteristics, dose ranges, routes of administration, and mechanisms of action.

Clinical Domains of Included Reviews	Clinical Indications per Domain	Summary of Overall Signal Detection per Domain	Count of Systematic Reviews with Meta-Analysis per Domain	Overall Characteristics of Included Studies and/or Populations in the Reviews*	Average Dose Range per Domain	Routes of Administration Adopted per Domain	Mechanism of Action Adopted per Domain
**Cardiology/Hematology**	1. Myocardial infarction 2. Cardiac surgery 3. Transfusion 4. Anticoagulation	One review is against, others are neutral to supportive	3	a. 40 RCTs b. sample size = 4100 c. males>females	100-300 mg/kg/day	Oral, IV	Antioxidant, anti-inflammatory, reduce vitamin K-dependent coagulation factors, prevent cardiac re-modelling
**Clinical Toxicology**	1. Poisoning from: a. Amatoxin (mushroom toxin) b. aluminum phosphide (pesticide) c. Sulphur mustard d. Rodenticides 2. Ototoxicity induced by: a. cisplatin b. noise c. aminoglycoside (including nephrotoxicity)	Supportive, neutral in some cases	7	a. 44 studies (15 RCTs) b. sample size around 3750 c. young, elderly or critically ill patients	1200-2700 mg/day	Oral, IV bolus and infusions, inhalation, intra/trans tympanic infusions	Antioxidant, Anti-inflammatory, Anti-apoptotic, hepatoprotective, neuroprotective, Mitochondrial protection, ROS scavenger, Neurotransmission effects (elevates glutamate), local clearance and targeted delivery (intra-tympanic)
**Dermatology**	Acne vulgaris and related conditions	Supportive, neutral in PCOS related acne	0	a.22 studies b. sample size around 2000 patients	1200 mg/day, 5% gel	Oral, topical, IV	Antioxidant, Anti-inflammatory, Anti-proliferative, Neurotransmission modulation, Antimicrobial, Anti-fibrotic and wound healing
**Endocrinology**	Diabetes and Insulin Resistance	Supportive	0	a.8 studies b. sample size around 230	600-2700 mg/day; infusion: 125 mg/kg/hour	Oral, IV	Antioxidant, Anti-inflammatory, Endothelial function preservation, Anti-apoptotic, Enhances antioxidant defense and insulin genes transcription, Improves lipid metabolism, Signaling & immune-modulation, Neuroprotective
**Geriatric Medicine**	Sarcopenic obesity, muscle weakness, Ageing hallmarks, cognitive decline	Supportive	0	a.Pilot studies b. sample size = 16 c. healthy older adults (>65 years old)	132 mg/kg/day	Oral	Antioxidant, Anti-inflammatory, Geno-toxicity reduction, Mitochondrial protection, Cellular protection, Physiologic regulation and positive metabolic effects
**Hepatology/ Gastroenterology**	1.Dengue fever induced liver injury 2. Hepatic diseases (i.e., non-alcoholic fatty liver disease) 3. Drug-induced (non-paracetamol) induced acute liver injury/ failure 4. Liver resection or transplant 5. Liver cancer 6. Pancreatitis	Majority (10 reviews) are inconclusive/neutral, one review is against, while 5 reviews are supportive	5	a.41 studies and 30 RCTs b. sample size around 6000 c. mixed adults and pediatrics	600-1200 mg/day or 150 mg/kg/day	Oral, IV (bolus and infusion) or added to graft solution in case of transplantation	Antioxidant, Anti-inflammatory, Vasodilatory (hemodynamic effects), Anti-apoptotic, Immuno-modulatory, Cellular/ mitochondrial protection, increase cytoprotective gene expression, insulin sensitizing
**Immunology**	1.Ulcerative Colitis 2. Rheumatoid Arthritis 3. Systemic Lupus Erythematosus	One review is neutral to against	1	a.4 RCTs b. sample size = 200	1200-2700 mg/day	Oral	Antioxidant, Anti-inflammatory, Immunomodulatory, Improves endothelial function
**Infectious Diseases**	1.Helicobacter eradication 2. Multi-drug resistant infections 3. Respiratory infections (i.e., RSV) and COVID 4. Urinary tract infections 5. Sepsis 6. Antibiotic efficacy 7. Tuberculosis	6 reviews are neutral and cautious while the rest are supportive	3	a.210 studies and 20 RCTs b. sample size around 27000	1200 mg/day	Oral, IV, Aerosol/ Nebulized, Topical/Nasal douche	Biofilm disruption, Antibacterial and synergistic effects, Antioxidant, Anti-inflammatory, Vasodilation, Immunomodulatory, Barrier protection, Vasoprotective, Antiviral, Antithrombotic, Glutamate release regulation (pain)
**Nephrology**	1.Contrast induced nephropathy 2. Acute kidney injury (i.e., after angiography or cardiac surgery) 3. Renal failure and hemodialysis 4. Cisplatin & Antimicrobial induced nephrotoxicity (i.e., vancomycin, colistin and amphotericin)	4 reviews are against, 2 are neutral and the rest are supportive	13	a. Around 400 RCTs and 110 studies are included in all reviews b. Sum of sample sizes= around 60000	1200-2400 mg/day	Oral, IV	Antioxidant, direct ROS scavenger, Vasodilation, Improve renal hemodynamics, Cardiac protective effects, Nephroprotective, Anti-inflammatory
**Neurology**	1.Neurodegenerative diseases 2.Traumatic Brain Injury 3. Parkinson Disease 4. Alzheimer’s Disease 5. Cognition 6. Autism 7. Diabetic peripheral neuropathy	2 reviews are neutral and the rest are supportive	1	a.12 studies and 7 RCTs b. pediatrics, adults and elderly patients c. sample size around 1200	1200-2700 mg/day	Oral, IV, Inhalation, Enteral	Antioxidant, Anti-inflammatory, Mitochondrial protection, Neuroprotective, Glutamate and NMDA receptor modulator, potential to mitigate amyloid/tau pathology (proteostasis support), Chelation of metals, Suppress nociceptive responses, Neurogenesis, Monoaminergic modulation, Synaptic plasticity restoration
**Oncology**	1.Cancer prevention 2. Ovarian cancer 3. Chemo resistance	2 reviews are supportive and one is neutral	0	Participants are healthy women or volunteers for pharmacokinetic studies	600-1800 mg/day	Oral	Conjugates estrogen quinones, complements with resveratrol, Cytoprotective, Anti-angiogenic, Antioxidant, ROS scavenger of therapy side effects, Immunomodulatory
**Oral Medicine**	1.Diseases/complications related to oral tissues, root canal and bones 2.Endodontic infections	Supportive	0	One RCT	25-200 mg/ml	Intra-canal	Antioxidant, Anti-inflammatory, Anti-biofilm, Antibacterial, Osteogenic, Anti-carcinogenic, Anti-mutagenic
**Pain Medicine**	1.Chronic pain 2.Perioperative use 3. Lower back pain	Supportive, 2 reviews were neutral to supportive	1	a.9 studies (5 RCTs) b. sample size around 1800	Oral:1200 mg/day; IV:150 mg/kg/day; IA: 300 mg/ 3 ml	Oral, IV, IA, Inhalation	Antioxidant, Anti-inflammatory, Cellular & mitochondrial protection, Neuroprotective, Neurotransmitter modulation, Anti-Apoptotic, Improves cartilage integrity
**Psychiatry**	1.Psychiatric, Mental and Neuropsychiatric disorders 2. Mood disorders: unipolar and bipolar depression 3. Cannabis, Substance and Alcohol Use Disorders 4. Grooming (i.e., Trichotillomania) and Obsessive Compulsive Disorders 5. Cocaine and Behavioral Addictions 6. Schizophrenia	3 reviews are against, 4 are neutral and the rest are supportive	9	a.79 RCTs b. Sample size around 12000	600-3000 mg/day	Mainly Oral (capsules, tablets and effervescent formulations); one review mentioned IV and Inhalation	Glutamatergic signaling modulation and homeostasis restoration, Antioxidant, Neuroprotective, Anti-inflammatory, Neurogenesis, Mitochondrial protection, Indirect dopaminergic modulation, Impact on ∆FosB protein, Anti-Apoptotic, NMDA regulation, Monoaminergic modulation, Synaptic plasticity
**Pulmonology**	1.Asthma 2. Cystic fibrosis 3. Lung injury 4. Chronic Bronchitis 5. COPD exacerbations 6. Pulmonary fibrosis	3 reviews are against and all the rest are supportive	9	a.44 RCTs and 59 studies b. Sample size around 17500 c. Majority are older males with smoking history	600-1800 mg/day	Oral, Inhaled	Antioxidant, Anti-inflammatory, Anti-fibrotic, Immunomodulatory, Anticoagulant, Vasoprotective
**Reproduction Medicine**	1.Male fertility and reproductive disruptions 2. Female fertility: PCOS, ovulation and hormones	Supportive	8	a.72 RCTs and 190 studies b. sample size around 10000 c. Majority young adults	600-1800 mg/day	Mainly oral, one review mentioned inhalation and IV	Antioxidant, Anti-inflammatory, Hormonal and Metabolic regulator, Insulin sensitizing, Anti-apoptotic, Mitochondrial protection, Anti-ferroptosis, Loosen cervical mucus and semen (mucolytic), Enhance sperm function and oocyte quality, Prevent viral invasion in testes
**Sports Medicine**	1.Recovery & muscle soreness/ physiology 2. Sports & muscle performance	Two reviews are supportive and the other two are neutral and against	3	a. 60 studies and 7 RCTs b. Majority males c. trained and untrained participants including surgical patients d. sample size around 1800	600-1200 mg/day; IV: 125–150 mg/kg/hour	Oral, IV	Antioxidant, Immunomodulatory, Anti-inflammatory, Cellular protection
***Summary:*** ***NAC repurposing spanning 17 clinical domains***	***> 85 distinct clinical indications evaluated***	***Majority of included reviews reported supportive conclusions***	***A total of 63 reviews represented the highest level of evidence captured***	***Approximately 724 RCTs involving around 150000 participants***	***Broad dosing regimens; 600–1200 mg/day frequently reported***	***Approximately 10 administration routes; oral and IV routes predominated***	***> 45 proposed mechanisms of action***

* Counts are review-level and may contain overlapping primary studies. COPD = Chronic Obstructive Pulmonary Disease, COVID: Coronavirus Disease, IA = Intra-articular, IV: Intravenous, NMDA = N-Methyl-D-Aspartate, PCOS: Polycystic Ovary Syndrome, RCT: Randomized Clinical Trial, ROS = Reactive Oxygen Species, RSV: Respiratory Syncytial Virus.

Most domains reported supportive signals for NAC, though neutral findings and 14 negative signals raise concerns about uncertainty. Contradictions were particularly evident in nephrology, psychiatry and hepatology, where large trial volumes yielded conflicting outcomes. Evidence certainty, assessed with GRADE (Grading of Recommendations Assessment, Development and Evaluation) in only one-quarter of meta-analyses, was predominantly low, raising questions about overall confidence. Since GRADE was not consistently reported across the included reviews, a combined matrix of AMSTAR-2, ROBIS, signal detection, and GRADE across domains was not constructed; instead, signal detection is summarized in Table 1 and in evidence maturity heatmap to avoid redundancy and maintain methodological clarity.

The distribution of systematic reviews and meta-analyses varied: five domains (toxicology, psychiatry, reproductive medicine, nephrology, and pulmonology) contained the highest concentration of high-level evidence, while several domains had none, indicating under exploration and opportunities for future research. Primary study characteristics were heterogeneous, with nephrology (~400 RCTs, ~ 60,000 patients) and psychiatry (~79 RCTs, ~ 12,000 patients) contributing the largest evidence bases, yet with mixed findings. While the infectious disease domain comprises a relatively large sample size (~27,000), the majority of these studies are derived from non-RCTs.

Dosing and administration were highly variable. The most frequent regimen was 1200 mg/day orally, though ranges extended from 600 mg/day oral to 300 mg/kg intravenous. Domain-specific routes (e.g., intra-tympanic, inhaled, intra-canal) were reported, highlighting variability in bioavailability, dosing and personalized delivery strategies. Duration of administration also varied widely, from at least 7 days to several months.

Mechanisms of action were diverse. Antioxidant and anti-inflammatory effects predominated, but domain-specific mechanisms emerged, such as biofilm disruption in infectious disease, hormonal regulation in reproductive medicine, and synaptic plasticity in psychiatry. Synergistic effects, including antibiotic potentiation and modulation of chemo-resistance, indicate NAC’s potential as an adjuvant therapy.

### 3.2. Reviews distribution

The included reviews originated from more than 25 countries worldwide. For clarity, only countries contributing more than one review are displayed in Fig 4. Countries accounting for 2% of the total were grouped together to reduce clutter and improve readability. China ranked first, accounting for 22% of reviews, followed by the United States (16%), with notable contributions from Italy, Iran, Australia, and India.

**Fig 4:**
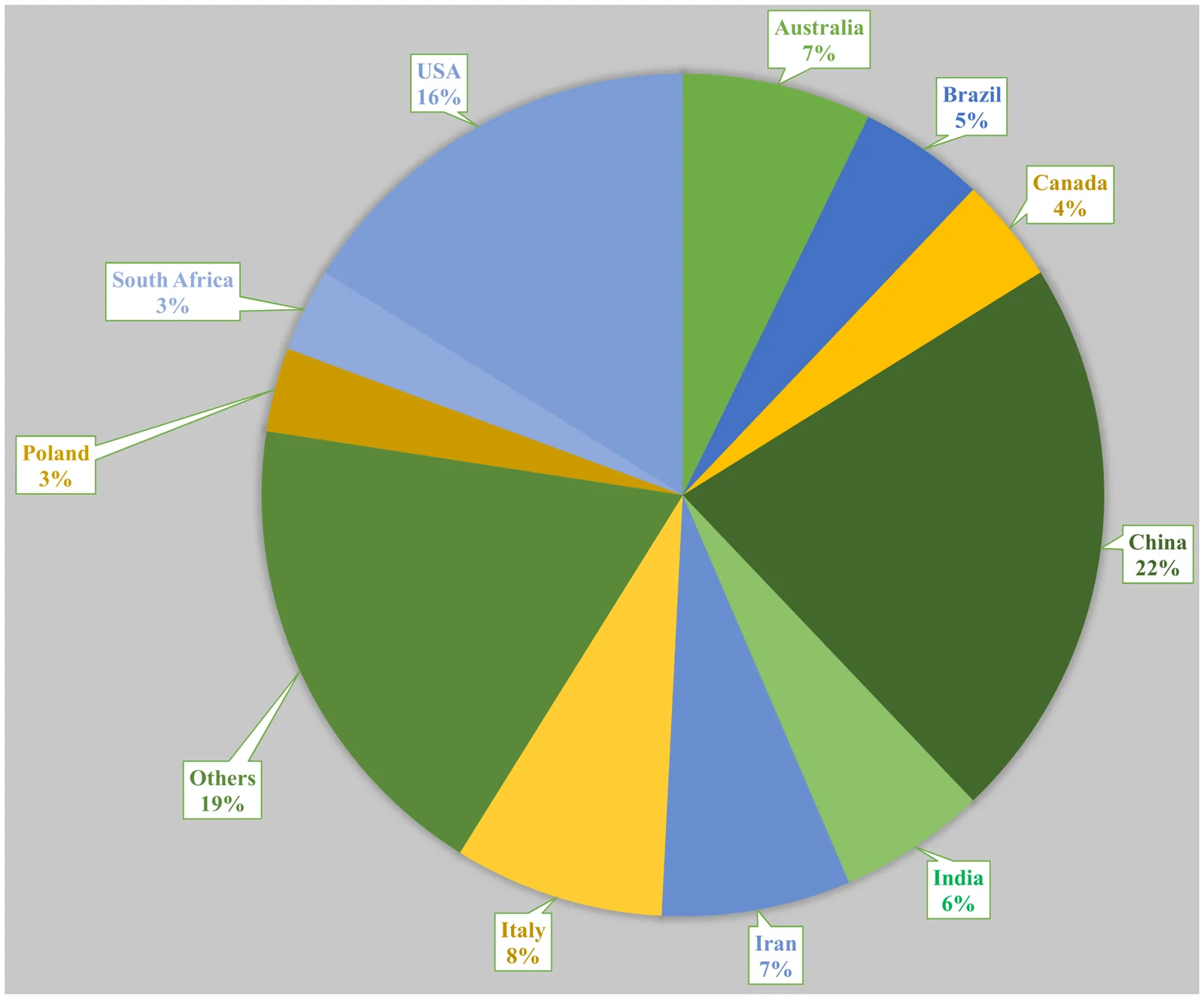
Geographic distribution of reviews. Pie chart showing the proportion (%) and the relative contribution of reviews by country. Percentages are based on the displayed data after excluding countries represented by a single study. “Others” group includes 10 countries: Spain, Taiwan, UK, Egypt, Singapore, The Netherlands, Tunisia, Greece, Japan and Jordan. These low-contribution countries are grouped for readability.

Fig 5 illustrates the distribution of reviews over time, with a line chart depicting both the overall growth and the evolution of individual clinical domains. The linear trend indicates sustained momentum, positioning NAC as a continuously evolving candidate in translational medicine. Publications have steadily increased since 2015, peaking between 2020 and 2022, reflecting heightened interest during the COVID-19 pandemic and broader exploration of NAC’s role in infectious and respiratory diseases. This surge also triggered growth in other domains during the same interval. In contrast, psychiatry and nephrology domains reached their peaks earlier, highlighting domain-specific trajectories.

**Fig 5:**
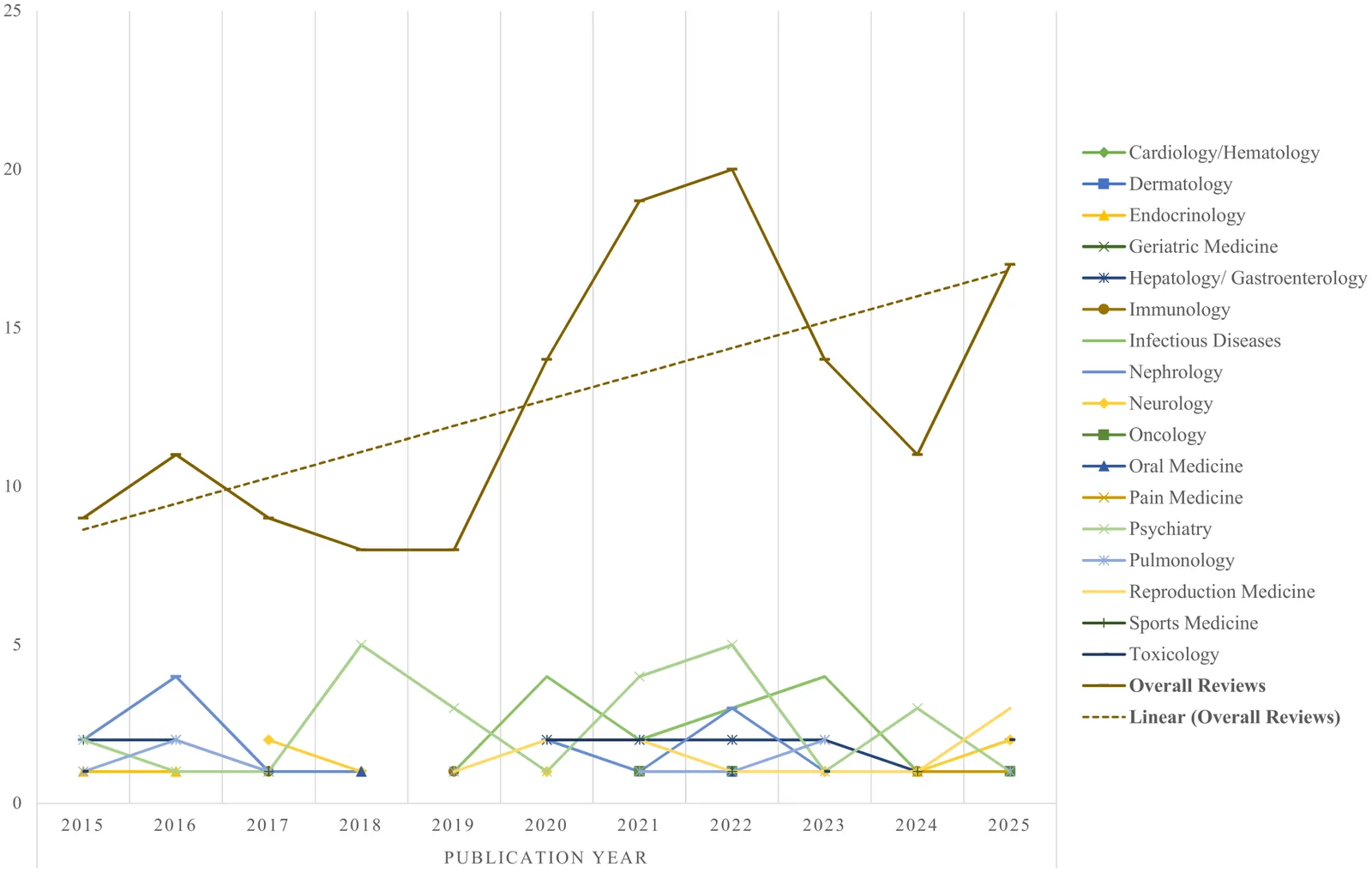
Temporal trends in publications. Line chart depicting the growth of NAC reviews over time. The figure shows overall publication volume and domain-specific trajectories. Domains with few published reviews naturally cluster near the baseline, reflecting the underexplored distribution of the mapped evidence.

### 3.3. Level of evidence distribution

Fig 6 illustrates marked heterogeneity in the evidence supporting NAC across clinical domains, presented as a heat map of review distribution by evidence level and type. Non-systematic reviews comprised primarily narrative formats, but also included critical, integrative, mini, and rapid reviews. Paradoxically, the majority of reviews fell at both extremes, meta-analyses and non-systematic reviews, yet yielded comparable levels of support (~60 studies each). Notably, umbrella reviews, which are considered a higher-level synthesis method in the literature, were absent across all domains, representing a critical gap given the abundance of studies and reviews. Less common review types tended to show stronger supportive signals for NAC repurposing, whereas the dominant types consistently reported >50% support. The map highlights numerous light squares, underscoring evidence gaps in dermatology, oral medicine, geriatrics, and oncology. Meta-analytic evidence was concentrated in nephrology, while infectious disease and psychiatry relied more heavily on narrative reviews. Nevertheless, nephrology, psychiatry, reproductive medicine, and pulmonology demonstrated higher concentrations of systematic and meta-analytic evidence, reflecting more mature research programs.

**Fig 6:**
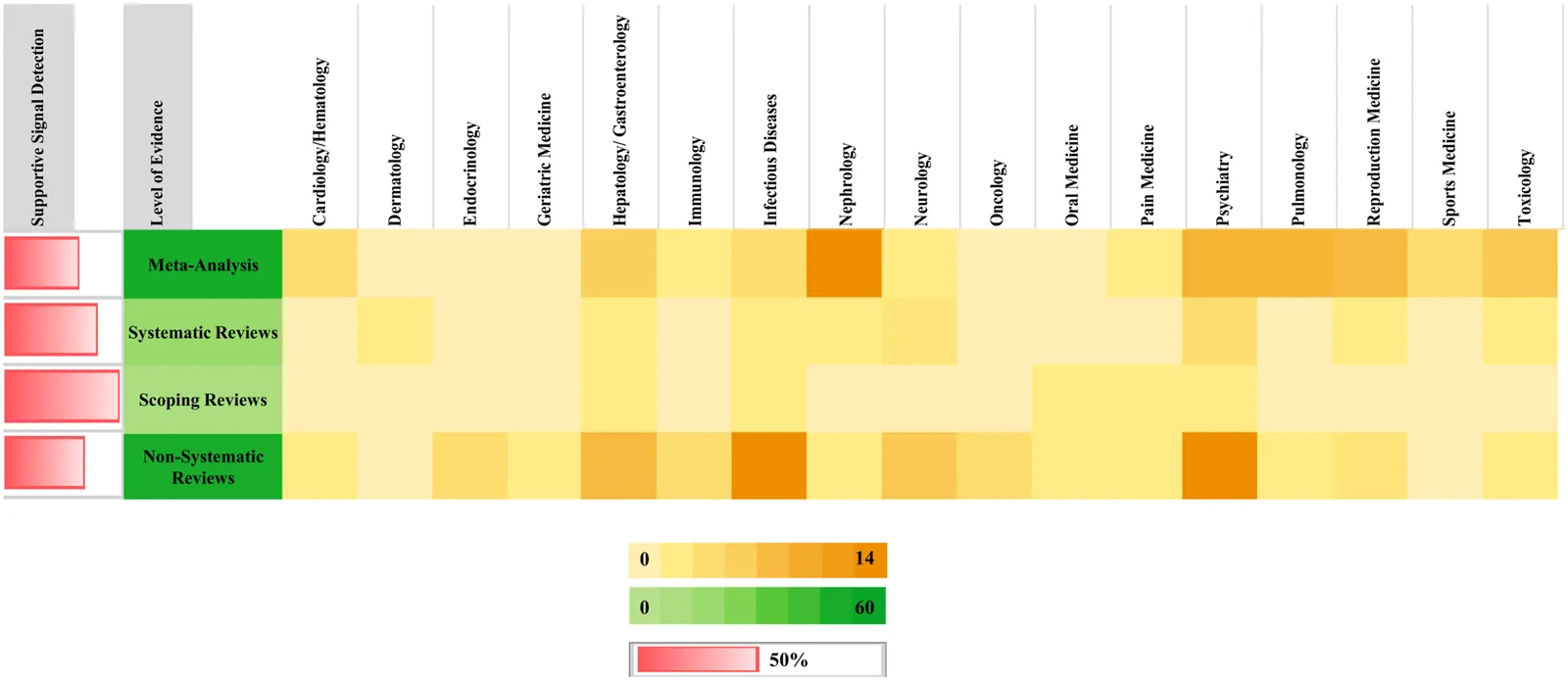
Evidence hierarchy. Heatmaps summarizing supportive signals (%) across evidence levels which are stratified by review type (frequency), highlighting heterogeneity in evidence strength and maturity across domains (frequency). Evidence levels are arranged in increasing order (bottom to top). Supportive percentages were calculated as the proportion of reviews within each evidence level that reported findings in favor, relative to the total number of reviews classified under that evidence level.

### 3.4. Reviews quality and risk of bias

Fig 7 highlights a critical methodological gap in NAC research, presenting two panels of horizontal stacked bars for comparative interpretation across clinical domains. The AMSTAR-2 ratings (left panel) reveal that critically low quality predominates, with high-quality reviews entirely absent. Specifically, 35 reviews were rated critically low, 29 low, and 10 moderate. The lack of publication bias assessment, largely due to small sample sizes, and the absence of protocol registration emerged as the most prevalent weaknesses, reducing transparency and reproducibility. Notably, the most extensively studied domains also scored the lowest in quality, underscoring uneven methodological rigor and limiting confidence while delaying the realization of NAC’s promise into clinical adoption.

**Fig 7:**
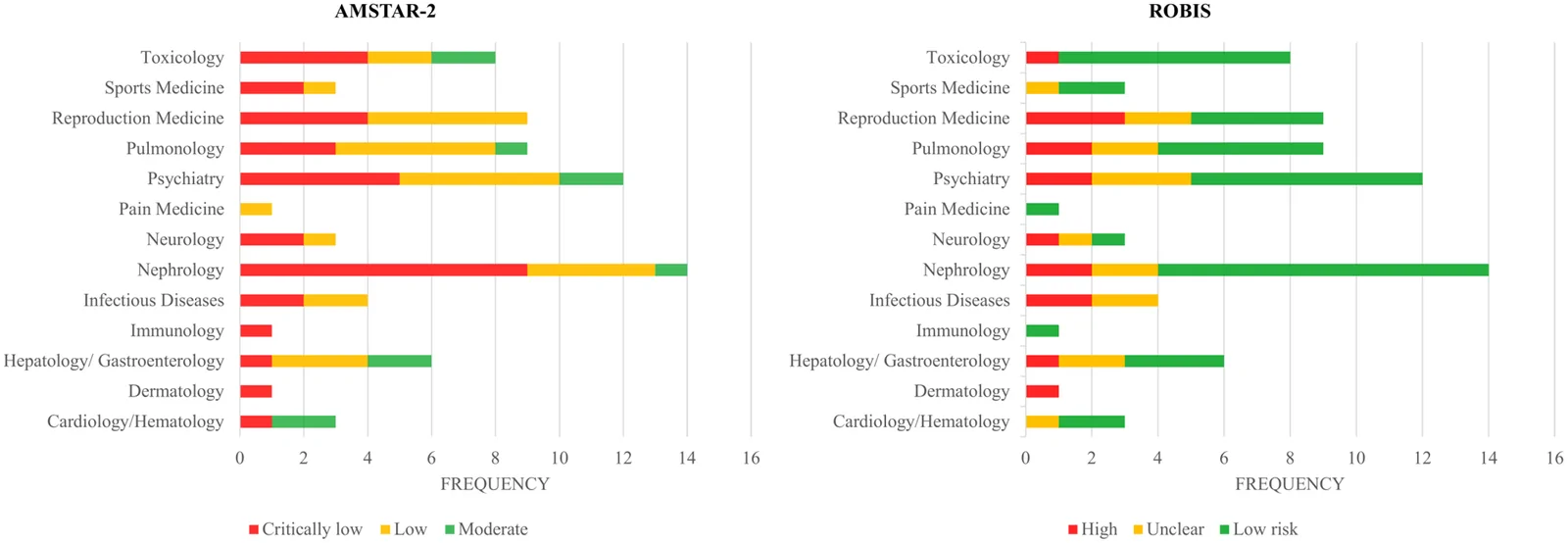
Methodological quality and risk of bias. Stacked bar chart of AMSTAR‑2 (left panel) and ROBIS (right panel) review assessments across domains (frequency). This dual appraisal across included systematic reviews guide interpretation of NAC’s repurposing potential. AMSTAR: A MeaSurement Tool to Assess systematic Reviews. ROBIS: Risk Of Bias In Systematic reviews.

In contrast, the ROBIS ratings (right panel) show that low risk was the predominant classification overall, including in domains that scored poorly on AMSTAR-2. The assessments showed 15 reviews at high risk of bias, 43 at low risk, and 16 unclear. The high ratings were mainly due to concerns in the data collection and appraisal domain (primarily the lack of risk-of-bias assessment), followed by the synthesis and findings domain (lack of consideration of bias assessments), and finally the identification and selection domain (issues with the search strategy). This discrepancy reflects variability in how reviews were conducted and reported, and highlights that methodological rigor and risk of bias are not interchangeable**.** A review may be methodologically weak (e.g., missing safeguards) yet still produce conclusions at low risk of bias (introduced bias), underscoring the importance of interpreting appraisal results in context.

Domain-specific patterns further emphasize insufficient evidence: dermatology, immunology, neurology, and sports medicine had fewer reviews, often of critically low quality, highlighting the need for stronger designs. Hepatology and neurology displayed mixed signals, with both critically low quality and unclear bias assessments.

### 3.5. Reviews overlap

Eight clinical domains containing systematic reviews or meta-analyses with comparable indications—prone to overlapping primary studies—were included in the CCA calculations. Fig 8 displays the distribution of domains in ascending order of percentage overlap, represented as dots. Categories passing CCA thresholds are color-coded, while dot size reflects the number of primary studies included, enabling meaningful interpretation.

**Fig 8:**
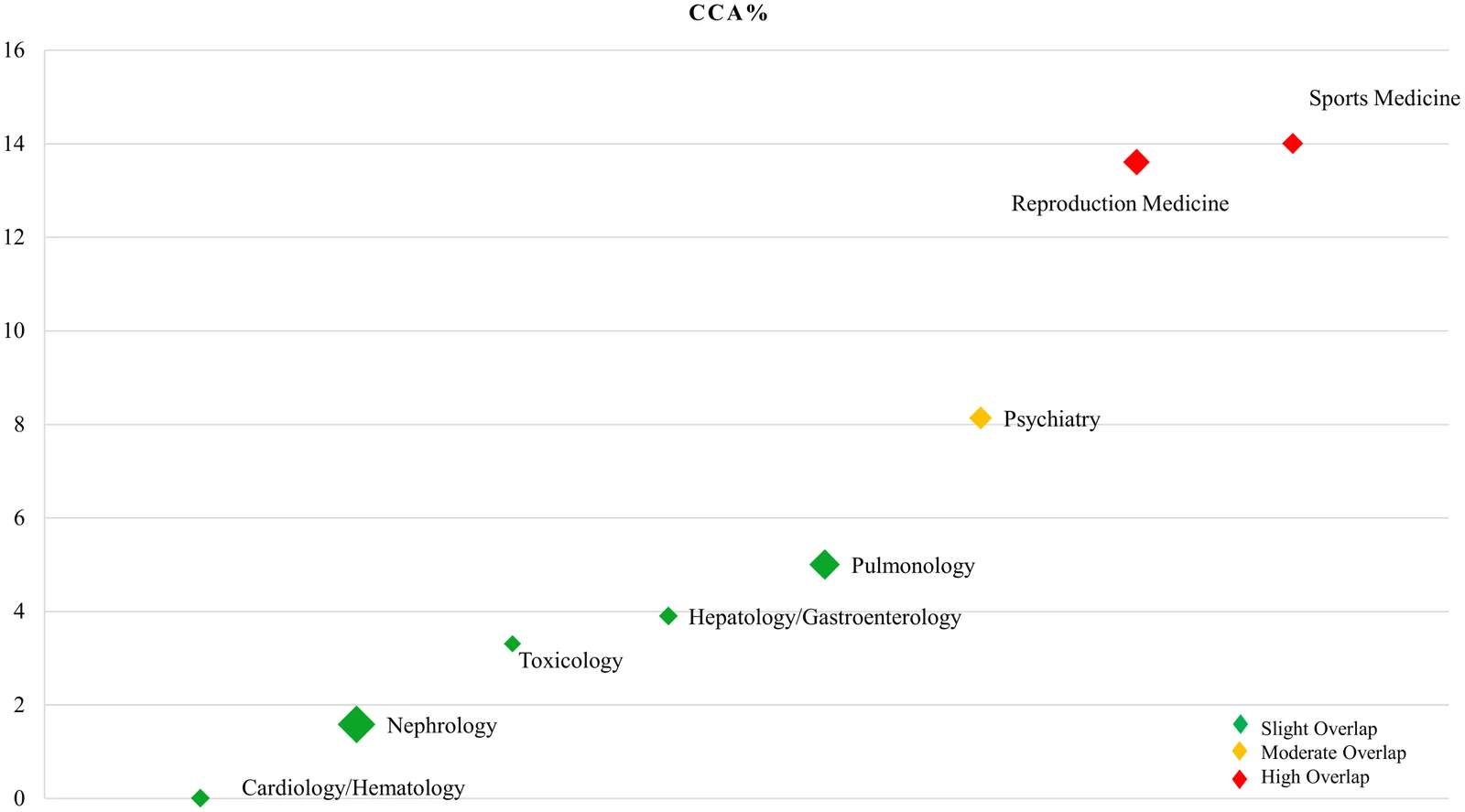
Overlap of reviews. Dot plot of corrected covered area (CCA%) showing redundancy among systematic reviews. Size of dots represents number of primary studies included in reviews of the corresponding domain. This analysis underscores the need for caution in interpreting results and highlights opportunities for more efficient research*.*

Sports medicine and reproduction medicine demonstrated high overlap (>12%), suggesting relatively consistent findings or repeated focus on similar outcomes, but also raising concern about evidence inflation and its impact on future clinical implementation. Psychiatry showed moderate overlap (≈8%), reflecting a growing yet heterogeneous and redundant evidence base. Nephrology, primarily focused on contrast-induced nephropathy, produced the largest dot, illustrating its extensive evidence base. Despite this, overlap was slight (<2%), positioning nephrology as a potential priority for future translational research. Other domains exhibited slight or low overlap, indicating fragmented, narrow, or highly variable evidence streams.

Subdomain analyses revealed more nuanced patterns. In cardiology, overall overlap was zero, but grouping by condition or population (across various domains) showed very high overlap for open cardiac surgery patients (25.7%) and moderate overlap (9.2%) for ST-elevation myocardial infarction (STEMI). Pulmonology revealed extreme variability: Chronic obstructive pulmonary disease (COPD) and chronic bronchitis had very high overlap (17.8%), while pulmonary fibrosis showed slight overlap (4.1%). Psychiatry subdomains—depression, schizophrenia, and substance use disorder—all exceeded 40% overlap, indicating very high redundancy. Reproductive medicine, when stratified by gender, showed >30% overlap for fertility indications, also considered very high. Toxicology revealed moderate overlap overall, but excluding the ototoxicity subdomain reduced overlap to 3.3% (slight). These findings demonstrate that some domains are inconsistent (low overlap across conditions), while others are oversaturated (high overlap within narrow subdomains).

## 4. Discussion

This overview illustrates NAC’s pleiotropic nature and highlights several clinical domains with promising signals, though the evidence remains inconclusive. Across indications, anticipated efficacy was consistently observed, yet most findings were supported only by moderate certainty and methodological quality. Additional barriers to translation identified in this scoping review include contradictory findings, methodological heterogeneity, and redundancy across reviews. Collectively, these results underscore NAC’s translational promise while emphasizing the need for more rigorous confirmatory trials and higher-quality evidence synthesis to strengthen confidence in its clinical utility.

These findings delineate domains where NAC suggests prospective relevance for clinical application versus those where it remains hypothesis-generating, underscoring NAC’s dual identity as both an established candidate in certain fields and an exploratory agent in others. Oncology, dermatology, endocrinology, geriatric, immunology, oral medicine, sports medicine, pain medicine, cardiology, and neurology domains reflect preliminary or inconsistent findings that warrant exploratory research rather than immediate clinical translation. Importantly, even domains with robust systematic reviews (e.g., nephrology) revealed contradictory outcomes, indicating that evidence volume does not equate to clinical consensus. Domains with supportive yet dispersed evidence are particularly suited for large-scale randomized controlled trials (RCTs) and meta-analyses to advance translational readiness. Collectively, these findings raise a pressing question regarding how clinicians should interpret NAC evidence when review volume is high but methodological quality remains low. Areas with low overlap but high clinical relevance should be prioritized for standardized trial designs to clarify NAC’s role. Even domains with low overall overlap revealed redundancy in specific scenarios, suggesting possible wasted research resources without advancing knowledge. This delays innovation and undermines confidence in NAC’s translational utility. High-overlap subdomains such as psychiatry, COPD, and reproductive medicine are particularly suited for umbrella reviews to synthesize redundant data into actionable guidance.

The reviews distribution reflects both strong Western clinical interest and significant Asian engagement, suggesting cross-cultural interest in NAC’s therapeutic potential. Smaller contributions from countries such as Tunisia, Jordan, Egypt, and Greece highlight emerging but underrepresented regions, underscoring the need for broader global research engagement. Importantly, the distribution indicates that interest in NAC—a low-cost, repurposable medicine—is not confined to resource-limited settings. The predominance of evidence from high-income countries, with sparse contributions from low-income countries, raises concerns about global generalizability and underscores the need for expanded involvement to ensure equitable translational relevance. The prominence of certain countries highlights regional differences in publication volume and research focus. While this scoping review cannot determine causal influences, future research may explore whether cultural, dietary, or genetic factors contribute to variations in NAC outcomes. Additionally, the COVID-era spike underscores NAC’s tendency to be rediscovered during emergencies, prompting discussion of whether NAC might be positioned as a repurposing candidate for future pandemics or multi-system crises.

### 4.1. Near-term candidates and contradictions

Several domains warrant deeper exploration to confirm existing indications or expand into new ones. For tested indications, higher levels of evidence are needed, while domains already supported by multiple systematic reviews and meta-analyses require trial refinement and resolution of heterogeneity to strengthen supportive signals. Domains with supportive findings and low overlap are highly suited for umbrella reviews and, in some cases, could inform future clinical decision-making*.*

Nephrology, pulmonology, psychiatry, reproductive medicine, and toxicology emerge as the clearest promising candidates for translational opportunities. This exploratory prioritization was based on a structured appraisal combining several criteria, rather than on a formal scoring framework with thresholds: (i) the relative volume of high-level evidence (systematic reviews with meta-analysis), (ii) the proportion of supportive signals within those reviews, (iii) methodological quality as assessed by AMSTAR-2 and ROBIS, and (iv) overlap patterns identified through CCA analysis. Domains meeting multiple criteria consistently were judged to hold the strongest translational potential, thereby guiding future research focus.

On the other hand, based on the findings of this review, nephrology, pulmonology, psychiatry and hepatology, despite abundant data, remain hindered by conflicting outcomes. Precision medicine approaches, including patient stratification and biomarker-guided analyses, are needed to resolve these contradictions.

Mechanisms of action of NAC across domains should be further validated and explored, with attention to novel targets and disease contexts informed by NAC’s biochemical properties. In COPD, trials using glutathione-generating antioxidants such as N-acetylcysteine, carbocysteine, and erdosteine have demonstrated reductions in exacerbations, though it remains unclear whether benefits derive primarily from antioxidant or mucolytic effects [163]. Importantly, dietary antioxidants have not shown clinical efficacy in COPD, underscoring the need to disentangle mechanistic pathways and identify domain-specific drivers of therapeutic response [163].

In otology, debate persists over whether pharmaceutical treatment of noise-induced hearing loss is a realistic option, underscoring the uncertainty of NAC’s role in auditory protection [164]. Similarly, in oral medicine, NAC-based mouthwashes did not alter the oral microbiome [165], yet demonstrated antibacterial activity by eliminating 8 of 12 species resistant to calcium hydroxide [166]. These findings illustrate the paradox of abundant mechanistic data but limited clarity in clinical outcomes. These contradictions emphasize the need for approaches to determine where NAC can be reliably deployed.

### 4.2. Sources of evidence variability and strategic directions

Variability in NAC signals likely reflects multiple interacting factors that require further validation and prioritization in future trials. These include mechanistic alignment with oxidative stress burden, disease stage dependency, limitations of surrogate biomarkers, heterogeneous patient populations, underpowered trials, and inconsistent outcome definitions. Additional contributors are dosage variability, differences in administration routes, blood–brain barrier penetration, distinctions between chronic and acute disease contexts, timing of interventions, and concomitant therapies.

Dosing strategies and routes of administration require systematic validation, particularly in relation to bioavailability thresholds, glutathione replenishment capacity, and redox burden. These factors may vary not only with disease pathophysiology but also with patient phenotypes characterized by high oxidative stress. Inconsistencies, where identical doses yield divergent outcomes, underscore the need to integrate pharmacogenomics insights, tissue penetration dynamics, and intervention timing. Advances in nanotechnology offer promising solutions, including systemic and targeted dendrimer–N-acetylcysteine (D-NAC) nanomedicines, which enhance delivery and tissue specificity [167]. Similarly, novel thiol-amides such as NAC-amide (AD4/NACA) are emerging as therapeutic candidates for neurodegenerative disorders [168], reflecting the possible translational potential of next-generation NAC formulations.

Variability in NAC outcomes may also be influenced by tolerability and side-effect profiles, which, although rarely emphasized, warrant consideration. Even at high doses, adverse events are uncommon, yet specific routes of administration pose risks [16]. Inhalational use can cause direct airway irritation and acidity in patients with reactive airways, while intravenous administration has been associated with anaphylactoid histamine release in approximately 10% of cases and, rarely, esophageal ulceration or severe esophagitis with oral administration [169,170]. Furthermore, NAC may interfere with concentrations of concomitant medications in psychiatric settings, complicating interpretation of therapeutic signals [171]. These factors highlight the importance of integrating safety, pharmacological interactions, and patient-specific vulnerabilities into future trial designs to ensure both efficacy and translational confidence.

Addressing these sources of variability through standardized trial designs and tailored pharmacotherapy approaches will be essential for informing future integration into clinical practice. NAC’s adjuvant role, enhancing the efficacy of antibiotics, chemotherapy, and immunotherapy, should also be evaluated. The central challenge is not simply whether NAC is effective, but rather where, for whom, and under what conditions it can be reliably deployed. Addressing this requires global collaboration, methodological rigor, and targeted therapeutic approaches.

### 4.3. Emerging opportunities and indications

Neurology, pain medicine, geriatrics, and oncology show supportive signals in this review but require stronger methodological consolidation. Expanding pilot studies in geriatrics is particularly promising, as NAC’s mitochondrial protection and putative role in sarcopenia warrant further investigation of its geroprotective potential. Moreover, assessing acrolein as a biomarker for stroke, dementia, renal failure, and Sjögren’s syndrome may highlight the possible importance of NAC derivatives as SH-group acrolein scavengers to preserve quality of life in the elderly [172].

Emerging indications further expand NAC’s therapeutic horizon. Evidence suggests that NAC, alongside melatonin, attenuates oxidative stress crosstalk through modulation of endoplasmic reticulum stress, positioning it as a plausible novel target for the prevention and treatment of hypertension and related diseases [173]. In dermatology, NAC has demonstrated potential in enhancing drug penetration across the nail plate, offering new strategies for managing onychomycosis [174]. Nutritional studies highlight its likely role as an antioxidant in maternal diets during pregnancy, with implications for mitigating developmental programming of chronic diseases [175].

At the cellular level, NAC has also been classified as a ferroptosis inhibitor, a pathway increasingly implicated in neurodegenerative disorders such as Alzheimer’s, Parkinson’s, Huntington’s disease, amyotrophic lateral sclerosis, multiple sclerosis, and Friedreich’s ataxia [176]. Preclinical evidence further supports NAC’s beneficial effects against obesity-associated complications, underscoring its potential relevance in metabolic disease [177]. Collectively, these emerging domains highlight NAC’s versatility and call for systematic exploration to clarify its role across diverse clinical contexts [178].

### 4.4. Future recommendations

Future studies may explore biomarkers (e.g., oxidative stress, glutamate levels, mitochondrial function) to identify responders versus non-responders. Future investigations must move beyond volume toward rigor and translation. Methodological standardization, particularly harmonizing endpoints, dosing strategies, and outcome definitions, will be essential to reduce heterogeneity across systematic reviews. Building an evidence-to-practice pipeline will require mechanisms such as consensus panels and guideline development to evaluate supportive signals and guide their translation into potential actionable recommendations. Future NAC studies and funding strategies should avoid repeating the same subdomain questions (e.g., COPD, depression) and instead expand into underexplored conditions to balance the evidence landscape. Funding may be particularly valuable in domains with promising signals but critically low-quality reviews, including dermatology and geriatrics, to strengthen translational readiness. Above all, future efforts must deliver high-quality, bias-mitigated reviews to strengthen reliability and inform NAC’s potential path toward practice.

Future research should also clarify the policy relevance of NAC repurposing. For example, in low- and middle-income countries, NAC’s affordability and accessibility could inform resource allocation decisions and guideline development. In high-income countries, by contrast, the challenge lies in critically appraising whether NAC’s use should extend beyond its established role in acetaminophen overdose. Explicitly addressing these differential implications will help align regulatory guidance and clinical practice frameworks with local needs.

### 4.5. Limitations

Despite the breadth and synthesis of this review, several limitations should be acknowledged. The most notable is its single-author design, which may raise concerns about bias. For example, inter-rater reliability for signal classification and critical appraisals could not be formally assessed. However, transparency at every step, including protocol pre-registration, adherence to reporting guidelines, use of predefined extraction tables, dual appraisal tools, overlap calculations, and provision of supplementary files, was intended to mitigate these drawbacks and enhance reproducibility.

Other limitations include reliance on a restricted set of databases, English-language reviews only—introducing language bias and limiting global generalizability—and a ten-year search window, chosen for feasibility and accessibility constraints but potentially narrowing coverage. Excluding grey literature further makes the synthesis prone to publication bias. However, the five databases ensured broad coverage across clinical and evidence-synthesis platforms, thereby minimizing the risk of missing relevant reviews. Reference-list screening was employed to reduce missed inclusions, particularly relevant given the recent rise of repurposing reviews.

As this study was designed as a scoping review of reviews, reliance on secondary evidence may propagate biases or omissions from the included reviews, limit appraisal of primary studies, and potentially miss emerging evidence. While appropriate for mapping the evidence landscape, this approach may dilute certainty and should be interpreted as overview signals rather than definitive trial-level conclusions.

Finally, limitations inherent to the included reviews must be noted: heterogeneous designs, conclusions, and populations reduce generalizability, while many domains remain immature, dominated by narrative and preclinical evidence. Publication bias was not formally assessed across reviews. However, critical appraisal revealed that many reviews did not report bias evaluation, which can be considered a limitation of the evidence base. In some cases, NAC was evaluated within multi-therapy approaches rather than as a single agent, further complicating interpretation. These constraints underscore the need for cautious appraisal of findings and highlight the importance of more rigorous, domain-specific investigations moving forward.

## 5. Conclusion

N-acetylcysteine emerges from this review as an adaptable and multifunctional molecule with notable translational potential. Despite the breadth of available evidence—spanning 140 studies across 17 clinical domains—the literature remains highly heterogeneous, inconsistent, redundant, and uncertain, which has delayed clinical implementation. NAC may thus be characterized as a candidate of high translational promise hindered by methodological weaknesses, underscoring the urgency for more rigorous investigation. NAC’s greatest translational promise may lie in its role as an adjuvant therapy and in stratified patient populations. Future research should address the gaps identified in this review, prioritizing the most advanced domains of evidence. The next step is the conduct of rigorously registered systematic reviews and meta-analyses, or umbrella reviews in mature domains, to consolidate signals and guide future trials. Such efforts will harmonize and clarify existing controversies, thereby facilitating evidence-based clinical implementation.

## Supporting information

S1 File PRISMA‑ScR checklist. Completed PRISMA‑ScR (Preferred Reporting Items for Systematic reviews and Meta‑Analyses extension for Scoping Reviews) checklist documenting adherence to reporting standards.
(PDF)

S2 File Comprehensive extraction table. Full data extraction table containing detailed information from all retained reviews.
(XLSX)

S3 File Critical appraisal and overlap scores. Individual scoring of all retained reviews using AMSTAR‑2 and ROBIS, with reasons for judgments where applicable. Includes an additional sheet grouping domains and subdomains, with detailed calculations of corrected covered area (CCA) for overlap analysis.
(XLSX)

S4 File List of retained reviews and references. Complete reference list of all reviews included in the scoping review.
(DOCX)

## Abbreviations

AMSTAR: A MeaSurement Tool to Assess systematic Reviews
CCA: Corrected Covered Area;
CDSR: Cochrane Database of Systematic Reviews
COPD: Chronic Obstructive Pulmonary Disease
COVID-19: Coronavirus Disease 2019
FDA: Food and Drug Administration
GRADE: Grading of Recommendations Assessment, Development and Evaluation
JBI: Joanna Briggs Institute
NAC: N-acetylcysteine
NAPQI: N-acetyl-p-benzoquinone imine
OSF: Open Science Framework
PCC: Population, Concept and Context
PRISMA-ScR: Preferred Reporting Items for Systematic reviews and Meta-Analyses for Scoping Reviews
RCT: Randomized Controlled Trial
ROBIS: Risk Of Bias In Systematic reviews
STEMI: ST-Segment Elevation Myocardial Infarction
TRIP: Turning Research Into Practice database
