# Supplementary material for: Repurposing landscape of N-acetylcysteine: A scoping review of reviews toward clinical translation
Source: PLoS One. 2026 Oct 9;21(10):e0360434. doi: 10.1371/journal.pone.0360434 (PMC13652227; doi:10.1371/journal.pone.0360434)
Supplement: Supplementary file 2 [file pone.0360434.s004.docx]

| **Study title** | **ID/Reference** |
| --- | --- |
| The 3,4-Quinones of Estrone and Estradiol Are the Initiators of Cancer whereas Resveratrol and N acetylcysteine Are the Preventers | [1] |
| N‑Acetyl Cysteine and Glutathione in Health and Cancer – Pharmacogenomics, Research, and Clinical Practice: Hypothesis and Review | [2] |
| N‑Acetyl Cysteine as a promising therapeutic approach in ovarian cancer: potential and perspectives | [3] |
| The impact of N- acetylcysteine on lactate, biomarkers of oxidative stress, immune response, and muscle damage: A systematic review and meta- analysis | [4] |
| N‑Acetyl‑L‑cysteine in human rheumatoid arthritis and its effects on nitric oxide (NO) and malondialdehyde (MDA): analytical and clinical considerations | [5] |
| The Potential Use of Metformin, Dipyridamole, N-Acetylcysteine and Statins as Adjunctive Therapy for Systemic Lupus Erythematosus | [6] |
| EfficacyofN-AcetylcysteineasanAdjuvantTherapyfor RheumatoidArthritis:ASystematicReviewand Meta-AnalysisofRandomizedControlledTrials | [7] |
| Role of N-acetylcysteine in liver injury due to dengue fever | [8] |
| N-acetylcysteine as an adjuvant therapy for Helicobacter pylori eradication | [9] |
| Rationale and evidence for the adjunctive use of  N-acetylcysteine in multidrug-resistant infections | [10] |
| Antibacterial and anti-inflammatory efficacy  of N-acetyl cysteine in endodontic treatment:  a scoping review | [11] |
| The effect of N-acetylcysteine on biofilms: Implications for the treatment of respiratory tract infections | [12] |
| N-Acetylcysteine’s Role in Sepsis and Potential Benefit in Patients With Microcirculatory Derangements | [13] |
| Novel Key Ingredients in Urinary Tract Health—The Role of D-mannose, Chondroitin Sulphate, Hyaluronic Acid, and N-acetylcysteine in Urinary Tract Infections (Uroial PLUS®) | [14] |
| Efficacy and Safety of N-Acetylcysteine for the Management of Chronic Pain in Adults: A Systematic Review and Meta-Analysis | [15] |
| Potential of N-acetylcysteine in the management of low back pain: a scoping review of studies in humans and animal models | [16] |
| Perioperative N-acetylcysteine: evidence and  indications | [17] |
| N-acetylcysteine as a treatment for amatoxin poisoning: a systematic review | [18] |
| Efficacy of intravenous N acetylcysteine as an  adjuvant therapy in the treatment of acute  aluminum phosphide Poisoning: a systematic  review and meta-analysis | [19] |
| ROLE OF N-ACETYL CYSTEINE IN RODENTICIDE  POISONING - A SYSTEMATIC REVIEW AND META ANALYSIS | [20] |
| N-Acetylcysteine as a treatment for sulphur mustard poisoning | [21] |
| Effect of N-acetyl-cysteine in prevention  of noise-induced hearing loss: a systematic review  and meta-analysis of randomized controlled trials | [22] |
| Intratympanic N-acetylcysteine in the  prevention of cisplatin-induced ototoxicity:  a systematic review and meta-analysis  of randomized controlled trials | [23] |
| Effect of N‐acetyl‐cysteine treatment on sensorineural hearing loss: a meta‐analysis | [24] |
| A systematic review and meta-analysis of the efficacy and safety of N-acetylcysteine in preventing aminoglycoside-induced ototoxicity: implications for the treatment of multidrug-resistant TB | [25] |
| The effect of N-acetylcysteine (NAC) on human cognition– A systematic review | [26] |
| N‑Acetylcysteine and Probenecid Adjuvant Therapy for Traumatic Brain Injury | [27] |
| Effect of N-Acetyl Cysteine as an Adjuvant Treatment in Alzheimer’s Disease | [28] |
| Effectiveness of N-acetylcysteine  in autism spectrum disorders:  A meta-analysis of randomized  controlled trials | [29] |
| Repositioning drugs for traumatic brain injury- N-acetyl cysteine and Phenserine | [30] |
| N-Acetyl-Cysteine: Modulating the Cysteine Redox Proteome in Neurodegenerative Diseases | [31] |
| Overview on the Effects of N-Acetylcysteine in Neurodegenerative Diseases | [32] |
| N-acetylcysteine use among patients undergoing cardiac surgery: A systematic review and meta-analysis of randomized trials | [33] |
| The Clinical Efficacy of N-Acetylcysteine in the Treatment of ST Segment Elevation Myocardial Infarction: A Meta-Analysis and Systematic Review | [34] |
| The effect of Vitamin E and N-acetyl cysteine on oxidative status and hemoglobin level in  transfusion-dependent thalassemia patients: A systematic review and meta-analysis | [35] |
| The effect of acetylcysteine on the prothrombin time and international normalized ratio: a narrative review | [36] |
| Asystematic review of N-acetylcysteine for treatment of acne vulgaris and acne-related associations and consequences: Focus on clinical studies | [37] |
| Oxidative Stress, Endothelial Dysfunction, and N-Acetylcysteine in Type 2 Diabetes Mellitus | [38] |
| A review on the possible molecular mechanism of action of N-acetylcysteine against insulin resistance and type-2 diabetes development | [39] |
| Role of N-Acetyl Cysteine in the Management of Diabetic Peripheral Neuropathy: A  Systematic Review | [40] |
| Efficacy and safety of acetylcysteine in‘‘non acetaminophen’’ acute liver failure: A meta-analysis  of prospective clinical trials | [41] |
| Oxidative Stress and Inflammation in Hepatic Diseases: Therapeutic Possibilities of N-Acetylcysteine | [42] |
| N‑acetylcysteine for non‑acetaminophen induced acute liver failure: A review | [43] |
| N-acetylcysteine for non-paracetamol drug induced liver injury: a systematic review | [44] |
| Current evidence for the use of N-acetylcysteine following liver resection | [45] |
| N-acetylcysteine in the Donor, Recipient,  or Both Donor and Recipient in Liver  Transplantation: A Systematic Review With  Meta-analysis and Trial Sequential Analysis | [46] |
| N-Acetylcysteine Use in Non Acetaminophen-Induced Acute Liver Failure | [47] |
| Clinical use of N-acetyl cysteine during liver transplantation: Implications  of oxidative stress and inflammation as therapeutic targets | [48] |
| Drug-Induced Liver Injury: Clinical Evidence of N-Acetyl Cysteine Protective Effects | [49] |
| N-Acetyl Cysteine Targets Hepatic Lipid Accumulation to Curb Oxidative Stress and Inflammation in NAFLD: AComprehensive Analysis of the Literature | [50] |
| N-acetyl cysteine versus standard of care for non-acetaminophen induced acute liver injury: a systematic review and meta-analysis | [51] |
| Use of N-acetyl-cysteine in the Perioperative Period of Liver Transplantation: A Scoping Review | [52] |
| N-acetylcysteine for non-paracetamol (acetaminophen)-related acute liver failure | [53] |
| Effectiveness of Prevention Strategies for Contrast-Induced Nephropathy: A Systematic Review and Meta-analysis | [54] |
| Effect of N- acetylcysteine on prevention  of contrast- associated acute kidney injury in patients with STEMI undergoing primary percutaneous coronary intervention: a systematic review and meta- analysis of randomised controlled trials | [55] |
| Efficacy of N-Acetylcysteine in Preventing Acute Kidney Injury After Cardiac Surgery: A Meta Analysis Study | [56] |
| Meta-analytic Techniques to Assess the Association Between N-acetylcysteine and Acute Kidney Injury After Contrast Administration: A Systematic Review and Meta-analysis | [57] |
| Latest Clinical Evidence About Effect of Acetylcysteine on Preventing Contrast-Induced Nephropathy in Patients Undergoing Angiography: A Meta-Analysis | [58] |
| The effect of N-acetylcysteine on the incidence of contrast-induced kidney injury: A systematic review and trial sequential analysis | [59] |
| The efficacy of N-acetylcysteine plus sodium bicarbonate in the prevention of contrast-induced nephropathy after cardiac catheterization and percutaneous coronary intervention: A meta-analysis of randomized controlled trials | [60] |
| An update in the utilization of N acetyl cysteine & vitamin C for tackling the oxidative stress in acute kidney injury secondary to robust sepsis: A Systematic Review | [61] |
| Prevalence of contrast nephropathy following the  administration of N-acetyl cysteine; a systematic  review and meta-analysis | [62] |
| N-Acetyl Cysteine Therapy Does Not Prevent Renal Failure in High-Risk Patients Undergoing Open-Heart Surgery | [63] |
| N-acetylcysteine for the prevention of contrast induced nephropathy in patients with pre-existing renal insufficiency or diabetes: a systematic review and meta-analysis | [64] |
| N-acetylcysteine versus ascorbic acid or N-acetylcysteine plus ascorbic acid in  preventing contrast-induced nephropathy: a meta-analysis | [65] |
| N‑Acetylcysteine: more than preventing contrast‑induced nephropathy in uremic patients—focus on the antioxidant  and anti‑inflammatory properties | [66] |
| N-Acetylcysteine’s Renoprotective Effect in  Cardiac Surgery: A Systematic Review and  Meta-Analysis | [67] |
| Role of N-Acetylcysteine to Prevent Contrast-Induced Nephropathy: A Meta-analysis | [68] |
| Effect of N-acetylcysteine on antimicrobials  induced nephrotoxicity: a meta-analysis | [69] |
| N-acetyl cysteine in the treatment of alcohol use disorder in patients with liver disease: Rationale for further research | [70] |
| N-acetylcysteine as an adjunctive treatment for bipolar depressionand major depressive disorder: a systematic review and meta-analysis of double-blind, randomized placebo-controlled trials | [71] |
| N-acetylcysteine as an adjunctive treatment for bipolar depression: a systematic review  and meta-analysis of randomized controlled trials | [72] |
| The effect of N-acetylcysteine on bipolar depression: a systematic review and meta-analysis of randomized controlled trials | [73] |
| N-acetylcysteine as a new prominent approach for treating psychiatric disorders | [74] |
| Oral N-acetylcysteine in the treatment of obsessive-compulsive disorder: A systematic review of the clinical evidence | [75] |
| A review of N-acetylcysteine in the treatment of grooming disorders | [76] |
| Exploring the potential link between ΔFosB and N-acetylcysteine in craving/relapse dynamics: can N-acetyl cysteine standout as a possible treatment candidate? | [77] |
| N-acetylcysteine for treating cocaine addiction – A systematic review | [78] |
| N-acetylcysteine for major mental disorders: a systematic review and meta-analysis of randomized controlled trials | [79] |
| Oral acetylcysteine for neuropsychiatric  disorders | [80] |
| N-acetyl cysteine in the treatment of cannabis use disorder: A systematic review of clinical trials | [81] |
| Effect of N-acetylcysteine on craving in substance use disorders (SUD): a meta-analysis of randomized controlled trials | [82] |
| The Potential of N‑acetyl Cysteine in Behavioral Addictions and Related Compulsive and Impulsive Behaviors and Disorders: a Scoping Review | [83] |
| The Potential of N‑Acetyl‑L‑Cysteine (NAC) in the Treatment of Psychiatric Disorders | [84] |
| N-Acetyl Cysteine in the Treatment of Obsessive Compulsive and Related Disorders: A Systematic Review | [85] |
| N-Acetyl Cysteine: A Potential Adjunctive Therapy in Psychiatric Disorder | [86] |
| N-acetylcysteine for schizophrenia: A systematic review and meta-analysis | [87] |
| N-acetylcysteine in the treatment of psychiatric disorders: Current status and future prospects | [88] |
| N-Acetylcysteine for the Treatment of Psychiatric Disorders: A Review of Current Evidence | [89] |
| Antipsychotic Augmentation with N-acetylcysteine for Patients with Schizophrenia | [90] |
| Improvement of cognitive function in schizophrenia with N-acetylcysteine: A theoretical review | [91] |
| Meta-analysis of randomised controlled  trials with N-acetylcysteine in the  treatment of schizophrenia | [92] |
| N-acetyl cysteine as a treatment for substance use cravings:A meta-analysis | [93] |
| N‑acetylcysteine in substance use disorder: a lesson from preclinical and clinical research | [94] |
| The neuroprotective effects of N-acetylcysteine in psychiatric and neurodegenerative disorders: From modulation of glutamatergic transmission to  restoration of synaptic plasticity | [95] |
| Targeting Redox Regulation to Treat Substance Use Disorder using N-acetylcysteine | [96] |
| The Potential of N-Acetylcysteine for Treatment of Trichotillomania, Excoriation Disorder, Onychophagia, and Onychotillomania: An Updated Literature Review | [97] |
| The effects of N-acetylcysteine on ovulation and sex hormones profile in women with polycystic ovary syndrome: a systematic review and meta-analysis | [98] |
| Efficacy of N-Acetylcysteine in Polycystic Ovary Syndrome: Systematic Review and Meta-Analysis | [99] |
| Comparison of the efficacy between NAC and metformin in treating PCOS patients: a meta analysis | [100] |
| A review of antioxidant N-acetylcysteine in addressing polycystic ovary syndrome | [101] |
| A Meta-Analysis of the Efficacy of L-Carnitine/L-Acetyl-Carnitine or N-Acetyl-Cysteine in Men With  Idiopathic Asthenozoospermia | [102] |
| Meta-analysis of the efficacy and safety of L-carnitine and N-acetyl cysteine monotherapy for male idiopathic infertility | [103] |
| Antioxidant effects of N-acetylcysteine on the male  reproductive system: A systematic review | [104] |
| N-acetyl-cysteine as adjuvant therapy in female infertility: a systematic review and meta-analysis | [105] |
| The role of N-acetyl-cysteine (NAC) orally daily on the sperm parameters and serum hormones in idiopathic infertile men: A systematic review and meta-analysis of randomised controlled trials | [106] |
| Therapeutic potential of carnitine and N-Acetyl-Cysteine supplementation on sperm parameters and pregnancy outcomes in idiopathic male  infertility: A systematic review and meta-analysis of randomized control trials | [107] |
| N-acetyl cysteine as a potential regulator  of SARS-CoV-2-induced male reproductive  disruptions | [108] |
| Biological Activities and Potential Oral Applications of N-Acetylcysteine: Progress and Prospects | [109] |
| A Review on Various Uses of N-Acetyl Cysteine | [110] |
| Aminireview on N-acetylcysteine: An old drug with new approaches | [111] |
| Performance and Side Effects of Supplementation with N-Acetylcysteine: A Systematic Review and Meta-Analysis | [112] |
| The effects of N- acetylcysteine on recovery biomarkers: A systematic review and meta- analysis of controlled trials | [113] |
| Efficacy of N-Acetylcysteine in preventing post-endoscopic retrograde cholangiopancreatography pancreatitis: a systematic review and meta-analysis | [114] |
| GlyNAC Supplementation Improves Glutathione Deficiency, Oxidative Stress, Mitochondrial Dysfunction, Inflammation, Aging Hallmarks, Metabolic Defects, Muscle Strength, Cognitive Decline, and Body Composition: Implications for Healthy Aging | [115] |
| N-Acetylcysteine in the Treatment of Acute Lung Injury:Perspectives and Limitations | [116] |
| Additive Effects of Glutathione in Improving Antibiotic Efficacy in HIV–M.tb Co-Infection in the Central Nervous System: A Systematic Review | [117] |
| N-Acetylcysteine and Hydrogen Sulfide in Coronavirus Disease 2019 | [118] |
| Effect of Orally Administered N-Acetylcysteine on Chronic Bronchitis: A Meta-analysis | [119] |
| Influence of N-acetylcysteine on chronic bronchitis or COPD exacerbations: a meta-analysis | [120] |
| The effect of N-acetylcysteine on exacerbations of chronic obstructive pulmonary disease: A meta-analysis and systematic review | [121] |
| N-acetylcysteine for prevention and treatment of COVID-19: Current state of evidence and future directions | [122] |
| Rationale for the use of N-acetylcysteine in both prevention and adjuvant therapy of COVID-19 | [123] |
| Where are we with the use of N-acetylcysteine  as a preventive and adjuvant treatment for  COVID-19? | [124] |
| COVID-19: The Potential Role of Copper and  N-acetylcysteine (NAC) in a Combination of Candidate Antiviral Treatments Against SARS-CoV-2 | [125] |
| Neuropsychiatric Manifestations of COVID-19 Disease and Post COVID Syndrome: The Role of N-acetylcysteine and Acetyl-L-carnitine | [126] |
| Molecular Mechanisms of N-Acetylcysteine in RSV Infections and Air Pollution-Induced Alterations: A Scoping Review | [127] |
| The role of N-acetyl cysteine and some other clinical  antidotes in the treatment of patients with COVID-19;  review of the current evidence | [128] |
| Therapeutic potential of N-acetyl cysteine (NAC) in preventing cytokine storm in COVID-19: review of current evidence | [129] |
| N-Acetyl Cysteine as an Adjunct in the Treatment of Tuberculosis | [130] |
| Impact of N-Acetyl Cysteine (NAC) on Tuberculosis (TB) Patients—A Systematic Review | [131] |
| The effects of N-acetyl cysteine on acute viral respiratory infections in humans: A rapid review | [132] |
| N-acetylcysteine efficacy in patients hospitalized with COVID-19 pneumonia: a systematic review and meta-analysis | [133] |
| Clinical efficacy of Chinese herbs for supplementing qi and activating blood circulation combined with N-acetylcysteine in the treatment of idiopathic pulmonary fibrosis: A systematic review and network meta-analysis | [134] |
| Pirfenidone, nintedanib and N-acetylcysteine for the  treatment of idiopathic pulmonary fibrosis: a  systematic review and meta-analysis | [135] |
| Efficacy of N-Acetylcysteine in Idiopathic Pulmonary Fibrosis : A Systematic Review and Meta-Analysis | [136] |
| Efficacy of N-acetylcysteine plus pirfenidone  in the treatment of idiopathic pulmonary  fibrosis: a systematic review and meta-analysis | [137] |
| The efficacy of N-acetylcysteine in chronic  obstructive pulmonary disease patients: a  meta-analysis | [138] |
| Systematic review and meta-analysis of the efficacy of  N-acetylcysteine in the treatment of acute exacerbation of chronic obstructive pulmonary disease | [139] |
| N-Acetylcysteine Role in Maintaining Renal Function in Cancer Patients with Cisplatin-Based Chemotherapy | [140] |

**References**

[1]	Cavalieri E, Rogan E. The 3 , 4-Quinones of Estrone and Estradiol Are the Initiators of Cancer whereas Resveratrol and N -acetylcysteine Are the Preventers. Int J Mol Sci 2021;22:8238. https://doi.org/10.3390/ijms22158238.

[2]	Morales-Borges RH, Gonzalez MJ, Duconge J, Minich DM. N-Acetyl Cysteine and Glutathione in Health and Cancer-Pharmacogenomics, research, and Clinical Practice: Hypothesis and Review. Altern Ther 2022;28.

[3]	Kindlon EA, Pidgeon GP. N-Acetyl Cysteine as a promising therapeutic approach in ovarian cancer : potential and perspectives. Acad Oncol 2025. https://doi.org/10.20935/AcadOnco7784.

[4]	Sadowski M, Zawieja E, Chmurzynska A. The impact of N- ­ acetylcysteine on lactate , biomarkers of oxidative stress , immune response , and muscle damage : A systematic review and meta- ­analysis. J Cell Mol Med 2024;28:e70198. https://doi.org/10.1111/jcmm.70198.

[5]	Tsikas D, Mikuteit M. N ‑ Acetyl ‑ L ‑ cysteine in human rheumatoid arthritis and its effects on nitric oxide ( NO ) and malondialdehyde ( MDA ): analytical and clinical considerations. Amino Acids 2022;54:1251–60. https://doi.org/10.1007/s00726-022-03185-x.

[6]	Tan MKX, Heng TYJ, Mak A. The Potential Use of Metformin, Dipyridamole, N-Acetylcysteine and Statins as Adjunctive Therapy for Systemic Lupus Erythematosus. Cells 2019;8:323. https://doi.org/10.3390/cells8040323.

[7]	He T, Ren K, Xiang L, Yao H, Huang Y, Gao Y. Efficacy of N-Acetylcysteine as an Adjuvant Therapy for Rheumatoid Arthritis: A Systematic Review and Meta-Analysis of Randomized Controlled Trials. Br J Hosp Med 2024;85:1–16. https://doi.org/10.12968/hmed.2024.0560.

[8]	Gupta M, Gupta S, Sood D, Gupta A, Jesrani G. Role of N-acetylcysteine in liver injury due to dengue fever. Trop Doct 2023:1–6. https://doi.org/10.1177/00494755231176317.

[9]	Fontes LES, Martimbianco ALC, Zanin C, Riera R. N-acetylcysteine as an adjuvant therapy for Helicobacter pylori eradication (Review). Cochrane Database Syst Rev 2019. https://doi.org/10.1002/14651858.CD012357.pub2.www.cochranelibrary.com.

[10]	Oliva A, Pallecchi L, Rossolini GM, Travaglino F, Zanatta P. Rationale and evidence for the adjunctive use of N-acetylcysteine in multidrug-resistant infections. Eur Rev Med Pharmacol Sci 2023;27:4316–25.

[11]	Abdulrab S, Mostafa N, Al-Maweri SA, Abada H, Halboub E, Alhadainy HA. Antibacterial and anti ‑ inflammatory efficacy of N ‑ acetyl cysteine in endodontic treatment : a scoping review. BMC Oral Health 2022;22:398. https://doi.org/10.1186/s12903-022-02433-6.

[12]	Blasi F, Page C, Rossolini GM, Pallecchi L, Matera MG, Rogliani P, et al. The effect of N -acetylcysteine on biofilms : Implications for the treatment of respiratory tract infections. Respir Med 2016;117:190–7. https://doi.org/10.1016/j.rmed.2016.06.015.

[13]	Chertoff J. N -Acetylcysteine ’ s Role in Sepsis and Potential Benefit in Patients With Microcirculatory Derangements. J Intensive Care Med 2017. https://doi.org/10.1177/0885066617696850.

[14]	Crocetto F, Balsamo R, Amicuzi U, Luca L De, Falcone A, Mirto BF, et al. Novel Key Ingredients in Urinary Tract Health — The Role of D-mannose, Chondroitin Sulphate, Hyaluronic Acid, and N-acetylcysteine in Urinary Tract Infections (Uroial PLUS®). Nutrients 2023;15:3573. https://doi.org/10.3390/nu15163573.

[15]	Mohiuddin M, Pivetta B, Gilron I, Khan JS. Efficacy and Safety of N-Acetylcysteine for the Management of Chronic Pain in Adults : A Systematic Review and Meta-Analysis. Pain Med 2021;0:1–12. https://doi.org/10.1093/pm/pnab042.

[16]	Sinigaglia G, Fortunato LM, Grillo ML, Partata WA. Potential of N-acetylcysteine in the management of low back pain : a scoping review of studies in humans and animal models. Brazilian J Med Biol Res 2025;58:e14382. https://doi.org/10.1590/1414-431X2025e14382.

[17]	Wilson PR, Bridges KH, Scofield M, Wilson SH. Perioper ative N-acetylcysteine : evidence and indications. Pain Manag 2024;14:385–96. https://doi.org/10.1080/17581869.2024.2388504.

[18]	Liu J, Chen Y, Gao Y, Walline JH, Lu X, Yu S, et al. N -acetylcysteine as a treatment for amatoxin poisoning : a systematic review. Clin Toxicol 2020. https://doi.org/10.1080/15563650.2020.1784428.

[19]	Shaker HO, Rageh OES, Alnajar M, Alshamaly NF, Abdelmaged WA, Abd-ElGawad M. Efficacy of intravenous N acetylcysteine as an adjuvant therapy in the treatment of acute aluminum phosphide Poisoning : a systematic review and meta-analysis. BMC Pharmacol Toxicol 2023;24. https://doi.org/10.1186/s40360-023-00699-2.

[20]	Valliappan S, Nadar P, Maruvada SAIS, Thulasi SM, Pallamparthy G, Manoharan S, et al. ROLE OF N-ACETYL CYSTEINE IN RODENTICIDE POISONING - A SYSTEMATIC REVIEW AND META- ANALYSIS. TPM 2025;32:1109–17.

[21]	Sawyer TW. N-Acetylcysteine as a treatment for sulphur mustard poisoning. Free Radic Biol Med J 2020;161:305–20. https://doi.org/10.1016/j.freeradbiomed.2020.09.020.

[22]	Chang P, Liu C, Hung S, Kang Y. Effect of N-acetyl-cysteine in prevention of noise-induced hearing loss : a systematic review and meta-analysis of randomized controlled trials. Arch Med Sci 2022;18:1535–41. https://doi.org/10.5114/aoms/109126 Copyright.

[23]	Tawalbeh M, Ibrahim RM, Al-saraireh T, Khreesha L, Al Rawashdeh B. Intratympanic N-acetylcysteine in the prevention of cisplatin-induced ototoxicity : a systematic review and meta-analysis of randomized controlled trials. BMC Pharmacol Toxicol 2025;26. https://doi.org/10.1186/s40360-024-00829-4.

[24]	Bai X, Wang M, Niu X, Yu H, Yeu J-X, Sun Y. Effect of N ‐ acetyl ‐ cysteine treatment on sensorineural hearing loss : a meta ‐ analysis. World J Otorhinolaryngol Head Neck Surg 2022;8:205–12. https://doi.org/10.1016/j.wjorl.2021.01.005.

[25]	Kranzer K, Elamin WF, Cox H, Seddon JA, Ford N, Drobniewski F. A systematic review and meta-analysis of the efficacy and safety of N-acetylcysteine in preventing aminoglycoside-induced ototoxicity : implications for the treatment of multidrug-resistant TB. Thorax 2015;0:1–8. https://doi.org/10.1136/thoraxjnl-2015-207245.

[26]	Skvarc DR, Dean OM, Byrne LK, Gray L, Lane S, Lewis M, et al. The effect of N-acetylcysteine (NAC) on human cognition – A systematic review. Neurosci Biobehav Rev 2017;78:44–56. https://doi.org/10.1016/j.neubiorev.2017.04.013.

[27]	Clark RSB, Empey PE, Kochanek PM, Bell MJ. N ‑ Acetylcysteine and Probenecid Adjuvant Therapy for Traumatic Brain Injury. Neurotherapeutics 2023;20:1529–37. https://doi.org/10.1007/s13311-023-01422-z.

[28]	Martínez SML, Moisés FPP, Bitzer-quintero OK, Ramírez-jirano J, Delgado-lara DLC, Trujillo IC, et al. Effect of N-Acetyl Cysteine as an Adjuvant Treatment in Alzheimer ’ s Disease. Brain Sci 2025;15. https://doi.org/10.3390/brainsci15020164.

[29]	Lee T, Lee K, Lee C, Lee H-C, Tam K-W, Loh E-W. Effectiveness of N -acetylcysteine in autism spectrum disorders : A meta-analysis of randomized controlled trials. Aust New Zeal J Psychiatry 2020;00. https://doi.org/10.1177/0004867420952540.

[30]	Hoffer BJ, Pick CG, Hoffer ME, Becker RE, Chiang Y, Greig NH. Repositioning drugs for traumatic brain injury - N-acetyl cysteine and Phenserine. J Biomed Sci 2017;24. https://doi.org/10.1186/s12929-017-0377-1.

[31]	Martinez-banaclocha M. N -Acetyl-Cysteine : Modulating the Cysteine Redox Proteome in Neurodegenerative Diseases. Antioxidants 2022;11. https://doi.org/10.3390/antiox11020416.

[32]	Tardiolo G, Bramanti P, Mazzon E. Overview on the Effects of N -Acetylcysteine in Neurodegenerative Diseases. Molecules 2018;23:3305. https://doi.org/10.3390/molecules23123305.

[33]	Pereira JEG, El Dib R, Braz LG, Escudero J, Hayes J, Johnston BC. N-acetylcysteine use among patients undergoing cardiac surgery : A systematic review and meta-analysis of randomized trials. PLoS One 2019;14:e0213862. https://doi.org/10.1371/journal.pone.0213862.

[34]	Jiang S-J, Huang C-H. The Clinical Efficacy of N-Acetylcysteine in the Treatment of ST Segment Elevation Myocardial Infarction: A Meta-Analysis and Systematic Review. Int Hear J 2021;62:142–7. https://doi.org/10.1536/ihj.20-519.

[35]	Haghpanah S, Hosseini-bensenjan M, Zekavat OR, Bordbar M, Karimi M, Ramzi M, et al. The effect of Vitamin E and N-acetyl cysteine on oxidative status and hemoglobin level in transfusion-dependent thalassemia patients: A systematic review and meta-analysis. Iran J Blood Cancer J 2023;15:22–35.

[36]	Nazar M, Kootstra-ros JE, Mian P, Touw DJ, Sturkenboom MGG. The effect of acetylcysteine on the prothrombin time and international normalized ratio : a narrative review. Clin Toxicol 2025;63:166–75. https://doi.org/10.1080/15563650.2025.2451642.

[37]	Mardani N, Mozafarpoor S, Goodarzi A, Nikkhah F. A systematic review of N-acetylcysteine for treatment of acne vulgaris and acne-related associations and consequences : Focus on clinical studies. Dermatol Ther 2021:e14915. https://doi.org/10.1111/dth.14915.

[38]	Li X, Zou J, Lin A, Chi J, Hao H, Chen H, et al. Oxidative Stress, Endothelial Dysfunction, and N-Acetylcysteine in Type 2 Diabetes Mellitus. Antioxid Redox Signal 2024;40. https://doi.org/10.1089/ars.2023.0524.

[39]	Lasram MM, Dhouib IB, Annabi A, Fazaa S El, Gharbi N. A review on the possible molecular mechanism of action of N-acetylcysteine against insulin resistance and type-2 diabetes development. Clin Biochem 2015;48:1200–8. https://doi.org/10.1016/j.clinbiochem.2015.04.017.

[40]	Emara SM, El-wakeel LM, Abdelsalam MM, Fahmy SF. Role of N-Acetyl Cysteine in the Management of Diabetic Peripheral Neuropathy : A Systematic Review. Arch Pharm Sci Ain Shams Univ 2024;8:455–68. https://doi.org/10.21608/aps.2024.319906.1195.

[41]	Hu J, Zhang Q, Ren X, Sun Z, Quan Q. Efficacy and safety of acetylcysteine in ‘“ non-acetaminophen ”’ acute liver failure : A meta-analysis of prospective clinical trials. Clin Res Hepatol Gastroenterol 2015. https://doi.org/10.1016/j.clinre.2015.01.003.

[42]	De Andrade KQ, Moura FA, Dos Santos JM, De Araujo ORP, Santos JC de F, Goulart MOF. Oxidative Stress and Inflammation in Hepatic Diseases : Therapeutic Possibilities of N -Acetylcysteine. Int J Mol Sci 2015;16:30269–308. https://doi.org/10.3390/ijms161226225.

[43]	Jiang SX, Hussaini T, Yoshida EM. N ‑ acetylcysteine for non ‑ acetaminophen induced acute liver failure : A review. Saudi J Gastroenterol 2022;28:85–91. https://doi.org/10.4103/sjg.sjg.

[44]	Chughlay MF, Kramer N, Spearman CW, Werfalli M, Cohen K. N-acetylcysteine for non-paracetamol drug- induced liver injury : a systematic review. Br J Clin Pharmacol 2016;81:1021–9. https://doi.org/10.1111/bcp.12880.

[45]	Kemp R, Mole J, Gomez D. Current evidence for the use of N-acetylcysteine following liver resection. ANZ J Surg 2017. https://doi.org/10.1111/ans.14295.

[46]	Jia D, Guo S, Jia Z, Gao Z, You K, Gong J, et al. N-acetylcysteine in the Donor , Recipient , or Both Donor and Recipient in Liver Transplantation : A Systematic Review With Meta-analysis and Trial Sequential Analysis. Transplantation 2023;00:1–15. https://doi.org/10.1097/TP.0000000000004597.

[47]	Mcpheeters CM, Vanarsdale VM, Weant KA. N-Acetylcysteine Use in Non- Acetaminophen-Induced Acute Liver Failure. Adv J 2016;38:183–9. https://doi.org/10.1097/TME.0000000000000116.

[48]	Ntamo Y, Ziqubu K, Chellan N, Nkambule BB, Nyambuya TM, Mazibuko-mbeje SE, et al. Clinical use of N-acetyl cysteine during liver transplantation : Implications of oxidative stress and inflammation as therapeutic targets. Biomed Pharmacother 2022;147:112638. https://doi.org/10.1016/j.biopha.2022.112638.

[49]	Ntamo Y, Ziqubu K, Chellan N, Nkambule BB, Nyambuya TM, Mazibuko-mbeje SE, et al. Drug-Induced Liver Injury : Clinical Evidence of N-Acetyl Cysteine Protective Effects. Oxid Med Cell Longev 2021;2021. https://doi.org/10.1155/2021/3320325.

[50]	Dludla P V, Nkambule BB, Mazibuko-mbeje SE, Nyambuya TM, Marcheggiani F, Cirilli I, et al. N-Acetyl Cysteine Targets Hepatic Lipid Accumulation to Curb Oxidative Stress and Inflammation in NAFLD : A Comprehensive Analysis of the Literature. Antioxidants 2020;9:1283. https://doi.org/10.3390/antiox9121283.

[51]	Shrestha DB, Budhathoki P, Sedhai YR, Adhikari A, Poudel A, Aryal B, et al. N-acetyl cysteine versus standard of care for non-acetaminophen induced acute liver injury : a systematic review and meta-analysis. Ann Hepatol 2021;24:100340. https://doi.org/10.1016/j.aohep.2021.100340.

[52]	Dos Santos FAM, Muniz GVC, Da Rocha MERT, Diógenes SFG, Tôrres DGB, De Lima CM, et al. Use of N-acetyl-cysteine in the Perioperative Period of Liver Transplantation : A Scoping Review. Brazilian J Transplant 2024;27:e4224. https://doi.org/10.53855/bjt.v27i1.587_ENG.

[53]	Jtp S, T N, RD T. N-acetylcysteine for non-paracetamol (acetaminophen)-related acute liver failure (Review). Cochrane Database Syst Rev 2020. https://doi.org/10.1002/14651858.CD012123.pub2.www.cochranelibrary.com.

[54]	Subramaniam RM, Suarez-Cuervo C, Wilson RF, Turban S, Zhang A, Sherrod C, et al. Effectiveness of Prevention Strategies for Contrast-Induced Nephropathy: A Systematic Review and Meta-analysis. Ann Intern Med 2016;164. https://doi.org/10.7326/M15-1456.

[55]	Guo Z, Liu J, Lei L, Xue Y, Liu L, Huang H, et al. Effect of N- ­ acetylcysteine on prevention of contrast- ­ associated acute kidney injury in patients with STEMI undergoing primary percutaneous coronary intervention : a systematic review and meta- ­ analysis of randomised controlled trials. BMJ Open 2020;10:e039009. https://doi.org/10.1136/bmjopen-2020-039009.

[56]	Mei M, Zhao H, Pan Q, Pu Y, Tang M, Shen B. Efficacy of N-Acetylcysteine in Preventing Acute Kidney Injury After Cardiac Surgery : A Meta- Analysis Study. J Investig Surg 2017. https://doi.org/10.1080/08941939.2016.1269853.

[57]	Magner K, Ilin JV, Clark EG, Kong JWY, Davis A, Hiremath S. Meta-analytic Techniques to Assess the Association Between N-acetylcysteine and Acute Kidney Injury After Contrast Administration A Systematic Review and Meta-analysis. JAMA Netw Open 2022;5:e2220671. https://doi.org/10.1001/jamanetworkopen.2022.20671.

[58]	Xie W, Liang X, Lin Z. Latest Clinical Evidence About Effect of Acetylcysteine on Preventing Contrast-Induced Nephropathy in Patients Undergoing Angiography : A Meta-Analysis. Angiology 2020. https://doi.org/10.1177/0003319720950162.

[59]	Wang N, Qian P, Kumar S, Yan TD, Phan K. The effect of N-acetylcysteine on the incidence of contrast-induced kidney injury : A systematic review and trial sequential analysis. Int J Cardiol 2016;209:319–27. https://doi.org/10.1016/j.ijcard.2016.02.083.

[60]	Zhao S, Zhong Z, Qi G, Tian W. The efficacy of N-acetylcysteine plus sodium bicarbonate in the prevention of contrast-induced nephropathy after cardiac catheterization and percutaneous coronary intervention : A meta-analysis of randomized controlled trials. Int J Cardiol 2016;221:251–9. https://doi.org/10.1016/j.ijcard.2016.07.086.

[61]	Kaur KK, Allahbadia G, Singh M. An update in the utilization of N-acetylcysteine & vitamin c for tackling the oxidative stress in acute kidney injury secondary to robust sepsis- A systematic review. J Clin Nephrol 2022;6:001–18. https://doi.org/10.29328/journal.jcn.1001084.

[62]	Moghadam MK, Nasri H. Prevalence of contrast nephropathy following the administration of N-acetyl cysteine ; a systematic review and meta-analysis. J Prev Epidemiol 2021;6:e34. https://doi.org/10.34172/jpe.2021.34.

[63]	Rababa AM, Deo S V, Altarabsheh SE, Caro J De, Abu Tarboush N, Alzoubi KH, et al. N-Acetyl Cysteine Therapy Does Not Prevent Renal Failure in High-Risk Patients Undergoing Open-Heart Surgery. Heart Surg Forum 2016;19. https://doi.org/10.1532/hsf.1424.

[64]	Kang X, Hu D, Li C, Ai Z, Peng A. N -acetylcysteine for the prevention of contrast- induced nephropathy in patients with pre-existing renal insufficiency or diabetes : a systematic review and meta-analysis. Ren Fail 2015;37:297–303. https://doi.org/10.3109/0886022X.2015.1012985.

[65]	Feng Y, Huang X, Li L, Chen Z. N-acetylcysteine versus ascorbic acid or N-acetylcysteine plus ascorbic acid in preventing contrast-induced nephropathy: a meta-analysis. Nephrology 2018;23. https://doi.org/10.1111/nep.13068.

[66]	Cepaityte D, Leivaditis K, Varouktsi G, Roumeliotis A, Roumeliotis S, Liakopoulos V. N ‑ Acetylcysteine : more than preventing contrast ‑ induced nephropathy in uremic patients — focus on the antioxidant and anti ‑ inflammatory properties. Int Urol Nephrol 2023;55:1481–92. https://doi.org/10.1007/s11255-022-03455-3.

[67]	Tan YK, Luo H, Kang GS, Teoh KLK, Kofidis T. N-Acetylcysteine ’ s Renoprotective Effect in Cardiac Surgery : A Systematic Review and Meta-Analysis. Ann Thorac Cardiovasc Surg 2022;28:138–45. https://doi.org/10.5761/atcs.oa.21-00132.

[68]	Loomba RS, Shah PH, Aggarwal S, Arora RR. Role of N-Acetylcysteine to Prevent Contrast-Induced Nephropathy : A Meta-analysis. Am J Ther 2016;23:e172–83.

[69]	Qiu X, Yang S, Zhang Y, Wang Q, Kong L, Zhou L. Effect of N-acetylcysteine on antimicrobials induced nephrotoxicity : a meta-analysis. BMC Nephrol 2025;26. https://doi.org/10.1186/s12882-025-04037-y.

[70]	Morley KC, Baillie A, Brink W Van Den, Chitty KE, Brady K, Back SE, et al. N-acetyl cysteine in the treatment of alcohol use disorder in patients with liver disease: Rationale for further research. Expert Opin Investig Drugs 2018;27:667–75. https://doi.org/10.1080/13543784.2018.1501471.N-acetyl.

[71]	Kishi T, Miyake N, Okuya M, Sakuma K, Iwata N. N-acetylcysteine as an adjunctive treatment for bipolar depression and major depressive disorder : a systematic review and meta-analysis of double-blind , randomized placebo-controlled trials. Psychopharmacology (Berl) 2020. https://doi.org/10.1007/s00213-020-05629-2.

[72]	Nery FG, Li W, DelBello MP, Welge JA. N-acetylcysteine as an adjunctive treatment for bipolar depression: a systematic review and meta-analysis of randomized controlled trials. Bipolar Disord 2021. https://doi.org/10.1111/bdi.13039.

[73]	Pittas S, Theodoridis X, Haidich A-B, Bozikas P-V, Papazisis G. The effect of N-acetylcysteine on bipolar depression : a systematic review and meta-analysis of randomized controlled trials. Psychopharmacology (Berl) 2021. https://doi.org/10.1007/s00213-021-05789-9.

[74]	Smaga I, Frankowska M, Filip M. N -acetylcysteine as a new prominent approach for treating psychiatric disorders. Br J Pharmacol 2021;178:2569–94. https://doi.org/10.1111/bph.15456.

[75]	Couto JP, Moreira R. Oral N-acetylcysteine in the treatment of obsessive-compulsive disorder: A systematic review of the clinical evidence José. Prog Neuropsychopharmacol Biol Psychiatry 2017. https://doi.org/10.1016/j.pnpbp.2018.06.005.

[76]	Braun TL, Patel V, Debord LC, Rosen T. A review of N-acetylcysteine in the treatment of grooming disorders. Int J Dermatol 2019. https://doi.org/10.1111/ijd.14371.

[77]	Arjmand S, Ilaghi M, Shafie M, Gobira PH, Grassi-oliveira R, Wegener G. Exploring the potential link between Δ FosB and N -acetylcysteine in craving / relapse dynamics : can N -acetylcysteine stand out as a possible treatment candidate ? Acta Neuropsychiatr 2025;37:e31. https://doi.org/10.1017/neu.2024.38.

[78]	Echevarria MAN, Reis TA, Capatti GR, Soares VS, Da Dilveira DX, Fidalgo TM. N-acetylcysteine for treating cocaine addiction – A systematic review. Psychiatry Res 2017. https://doi.org/10.1016/j.psychres.2017.02.024.

[79]	Zheng W, Zhang Q., Cai D, Yang X, Qiu Y, Ungvari GS, et al. N -acetylcysteine for major mental disorders : a systematic review and meta-analysis of randomized controlled trials. ACTA Psychiatr Scand 2018. https://doi.org/10.1111/acps.12862.

[80]	Racz R, Sweet B V., Sohoni P. Oral acetylcysteine for neuropsychiatric disorders. Am J Heal Pharm 2015;72. https://doi.org/10.2146/ajhp140732.

[81]	Sharma R, Tikka SK, Bhute AR, Bastia BK. N-acetyl cysteine in the treatment of cannabis use disorder : A systematic review of clinical trials. Addict Behav 2022;129:107283. https://doi.org/10.1016/j.addbeh.2022.107283.

[82]	Cuocina M, Aiello G, Cutrufelli P, Rampello M, Rapisarda L, Rodolico A, et al. Effect of N-acetylcysteine on craving in substance use disorders ( SUD ): a meta-analysis of randomized controlled trials. Front Pharmacol 2024. https://doi.org/10.3389/fphar.2024.1462612.

[83]	Greenberg NR, Farhadi F, Kazer B, Potenza MN, Angarita GA. The Potential of N ‑ acetyl Cysteine in Behavioral Addictions and Related Compulsive and Impulsive Behaviors and Disorders : a Scoping Review. Curr Addict Reports 2022. https://doi.org/10.1007/s40429-022-00446-3.

[84]	Bradlow RCJ, Berk M, Kalivas PW, Back SE, Kanaan RA. The Potential of N ‑ Acetyl ‑ L ‑ Cysteine ( NAC ) in the Treatment of Psychiatric Disorders. CNS Drugs 2022;36:451–82. https://doi.org/10.1007/s40263-022-00907-3.

[85]	Oliver G, Dean O, Camfield D, Blair-west S, Ng C, Berk M, et al. N-Acetyl Cysteine in the Treatment of Obsessive Compulsive and Related Disorders : A Systematic Review. Clin Psychopharmacol Neurosci 2015;13:12–24. https://doi.org/10.9758/cpn.2015.13.1.12.

[86]	Khoja E, Erbaş O. N-Acetyl Cysteine : A Potential Adjunctive Therapy in Psychiatric Disorders. J Exp Basic Med Sci 2024;5:180–4. https://doi.org/10.5606/jebms.2024.1088.

[87]	Kishi T, Sakuma K, Hatano M, Iwata N. N -acetylcysteine for schizophrenia : A systematic review and meta-analysis. Psychiatry Clin Neurosci 2023;77:119–21. https://doi.org/10.1111/pcn.13502.

[88]	Minarini A, Ferrari S, Galletti M, Giambalvo N, Perrone D, Rioli G, et al. N-acetylcysteine in the treatment of psychiatric disorders : Current status and future prospects. Expert Opin Drug Metab Toxicol 2016. https://doi.org/10.1080/17425255.2017.1251580.

[89]	Ooi SL, Green R, Pak SC. N-Acetylcysteine for the Treatment of Psychiatric Disorders : A Review of Current Evidence. Biomed Res Int 2018;2018. https://doi.org/10.1155/2018/2469486.

[90]	Andrade C. Antipsychotic Augmentation With N-Acetylcysteine for Patients With Schizophrenia. J Clin Psychiatry 2022;83:22f14664. https://doi.org/10.4088/JCP.22f14664.

[91]	Yolland COB, Phillipou A, Castle DJ, Neill E, Hughes ME, Galletly C, et al. Improvement of cognitive function in schizophrenia with N-acetylcysteine : A theoretical review. Nutr Neurosci 2018. https://doi.org/10.1080/1028415X.2018.1478766.

[92]	Yolland COB, Hanratty D, Neill E, Rossell SL, Berk M, Dean OM, et al. Meta-analysis of randomised controlled trials with N -acetylcysteine in the treatment of schizophrenia. Aust New Zeal J Psychiatry 2019;00. https://doi.org/10.1177/0004867419893439.

[93]	Winterlind EL, Malone SG, Setzer MR, Murphy MA, Saunders D, Gray JC. N-acetylcysteine as a treatment for substance use cravings : A meta-analysis. Addict Biol 2025;29:e70001. https://doi.org/10.1111/adb.70001.

[94]	Smaga I, Frankowska M, Filip M. N ‑ acetylcysteine in substance use disorder : a lesson from preclinical and clinical research. Pharmacol Reports 2021;73:1205–19. https://doi.org/10.1007/s43440-021-00283-7.

[95]	Chakraborty S, Rao S, Tripathi SJ. The neuroprotective effects of N-acetylcysteine in psychiatric and neurodegenerative disorders: From modulation of glutamatergic transmission to restoration of synaptic plasticity. Neuropharmacology 2025. https://doi.org/1 0.1 01 6/ j.neuropharm.2025.1 1 0527.

[96]	Womersley JS, Townsend DM, Kalivas PW, Uys JD. Targeting Redox Regulation to Treat Substance Use Disorder using N-acetylcysteine. Eur J Neurosci 2019;50:2538–51. https://doi.org/10.1111/ejn.14130.

[97]	Lee DK, Lipner SR. The Potential of N -Acetylcysteine for Treatment of Trichotillomania , Excoriation Disorder , Onychophagia , and Onychotillomania : An Updated Literature Review. Int J Environ Res Public Health 2022;19:6370. https://doi.org/10.3390/ijerph19116370.

[98]	Asl ZS, Parastouei K, Eskandari E. The effects of N-acetylcysteine on ovulation and sex hormones profile in women with polycystic ovary syndrome : a systematic review and meta-analysis. Br J OfNutrition 2023;130:202–10. https://doi.org/10.1017/S0007114522003270.

[99]	Viña I, Viña JR, Carranza M, Mariscal G. Efficacy of N -Acetylcysteine in Polycystic Ovary Syndrome : Systematic Review and Meta-Analysis. Nutrients 2025;17:284. https://doi.org/10.3390/nu17020284.

[100]	Song Y, Wang H, Huang H, Zhu Z. Comparison of the efficacy between NAC and metformin in treating PCOS patients : a meta- analysis. Gynecol Endocrinol 2019. https://doi.org/10.1080/09513590.2019.1689553.

[101]	Yifu P. A review of antioxidant N-acetylcysteine in addressing polycystic ovary syndrome syndrome. Gynecol Endocrinol 2024;40. https://doi.org/10.1080/09513590.2024.2381498.

[102]	Wei G, Zhou Z, Cui Y, Huang Y, Wan Z, Che X, et al. A Meta-Analysis of the Efficacy of L-Carnitine / L-Acetyl-Carnitine or N-Acetyl-Cysteine in Men With Idiopathic Asthenozoospermia. Am J Men’s Heal 2021. https://doi.org/10.1177/15579883211011371.

[103]	Ma X, Yang Y, Liu S, Cui Y, Wu J. Meta-analysis of the efficacy and safety of L-carnitine and N-acetylcysteine monotherapy for male idiopathic infertility. Rev Int Androl 2025 2025;23. https://doi.org/10.22514/j.androl.2025.004.

[104]	Ghafarizadeh A, Malmir M, Noreini SN, Faraji T. Antioxidant effects of N-acetylcysteine on the male reproductive system : A systematic review. Andrologia 2020;00:e13898. https://doi.org/10.1111/and.13898.

[105]	Devi N, Boya C, Chhabra M, Bansal D. N -acetyl-cysteine as adjuvant therapy in female infertility : a systematic review and meta-analysis. J Basic Clin Physiol Pharmacol 2020. https://doi.org/10.1515/jbcpp-2020-0107.

[106]	Zhou Z, Cui Y, Zhang X, Zhang Y. The role of N-acetyl-cysteine ( NAC ) orally daily on the sperm parameters and serum hormones in idiopathic infertile men : A systematic review and meta-analysis of randomised controlled trials. Andrologia 2021;53:e13953. https://doi.org/10.1111/and.13953.

[107]	Ranneh Y, Hamsho M, Fadel A, Osman HMA, Ali EW, Kambal NHM. Therapeutic potential of carnitine and N-Acetyl-Cysteine supplementation on sperm parameters and pregnancy outcomes in idiopathic male infertility : A systematic review and meta-analysis of randomized control trials. Reprod Breed 2025;5:74–83. https://doi.org/10.1016/j.repbre.2025.02.002.

[108]	Sengupta P, Dutta S. N ‑ acetyl cysteine as a potential regulator of SARS ‑ CoV ‑ 2 ‑ induced male reproductive disruptions. Middle East Fertil Soc J 2022;27. https://doi.org/10.1186/s43043-022-00104-8.

[109]	Pei Y, Liu H, Yang Y, Yang Y, Jiao Y, Tay FR, et al. Biological Activities and Potential Oral Applications of N-Acetylcysteine : Progress and Prospects. Oxid Med Cell Longev 2018;2018. https://doi.org/10.1155/2018/2835787.

[110]	Mokhtari V, Afsharian P, Shahhoseini M, Kalantar SM, Moini A. A Review on Various Uses of N-Acetyl Cysteine. Cell J 2017;19:11–7.

[111]	Dhouib IE, Annabi A, Jallouli M, Elfazaa S, Lasram MM. A minireview on N-acetylcysteine: An old drug with new approaches. Life Sci 2016. https://doi.org/10.1016/j.lfs.2016.03.003.

[112]	Rhodes K, Braakhuis A. Performance and Side Effects of Supplementation with N -Acetylcysteine : A Systematic Review and Meta-Analysis. Sport Med 2017. https://doi.org/10.1007/s40279-017-0677-3.

[113]	Nejati M, Dehghan P, Jamilian P, Zarezadeh M. The effects of N- ­ acetylcysteine on recovery biomarkers : A systematic review and meta- ­ analysis of controlled trials. J Food Biochem 2022;46:e14116. https://doi.org/10.1111/jfbc.14116.

[114]	Hormati A, Mousavi A, Shojaei S, Moghtadaei A, Bordbar S, Alemi H, et al. Efficacy of N-Acetylcysteine in preventing post-endoscopic retrograde cholangiopancreatography pancreatitis : a systematic review and meta-analysis. BMC Gastroenterol 2025;25:783. https://doi.org/10.1186/s12876-025-04382-0.

[115]	Sekhar R V. GlyNAC Supplementation Improves Glutathione Deficiency, Oxidative Stress, Mitochondrial Dysfunction , Inflammation , Aging Hallmarks , Metabolic Defects , Muscle Strength , Cognitive Decline , and Body Composition : Implications for Healthy Aging. J Nutr 2021;151:3606–16. https://doi.org/10.1093/jn/nxab309.

[116]	Mokra D, Porvaznik I, Mokry J. N-Acetylcysteine in the Treatment of Acute Lung Injury : Perspectives and Limitations. Int J Mol Sci 2025;26:2657. https://doi.org/10.3390/ijms26062657.

[117]	Nabipur L, Mouawad M, Venketaraman V. Additive Effects of Glutathione in Improving Antibiotic Efficacy in HIV – M . tb Co-Infection in the Central Nervous System : A Systematic Review. Viruses 2025;17. https://doi.org/10.3390/v17010127.

[118]	Bourgonje AR, Offringa AK, Eijk LE Van, Abdulle AE, Hillebrands J, Voort PHJ Van Der, et al. N-Acetylcysteine and Hydrogen Sulfide in Coronavirus Disease 2019. ANTIOXIDANTS REDOX Signal Vol 2021;35:1207–25. https://doi.org/10.1089/ars.2020.8247.

[119]	Wei J, Pang C, Han J, Yan H. Effect of Orally Administered N -Acetylcysteine on Chronic Bronchitis : A Meta-analysis. Adv Ther 2019. https://doi.org/10.1007/s12325-019-01111-4.

[120]	Cazzola M, Calzetta L, Page C, Jardim J, Chuchalin AG, Rogliani P, et al. Influence of N -acetylcysteine on chronic bronchitis or COPD exacerbations : a meta-analysis. Eur Respir Rev 2015;24:451–61. https://doi.org/10.1183/16000617.00002215.

[121]	Fowdar K, Chen H, He Z, Zhang J, Zhong X, Zhang J, et al. The effect of N-acetylcysteine on exacerbations of chronic obstructive pulmonary disease : A meta-analysis and systematic review. Hear Lung 2017. https://doi.org/10.1016/j.hrtlng.2016.12.004.

[122]	Izquierdo-Alonso JL, Pérez-Rial S, Rivera CG, Peces-Barba G. N-acetylcysteine for prevention and treatment of COVID-19: Current state of evidence and future directions. J Infect Public Health 2020;15:1477–83. https://doi.org/10.1016/j.jiph.2022.11.009.

[123]	Flora S De, Balansky R, La Maestra S. Rationale for the use of N -acetylcysteine in both prevention and adjuvant therapy of COVID-19. The FASEB Journal 2020;34:13185–93. https://doi.org/10.1096/fj.202001807.

[124]	Marco FDI, Foti G, Corsico AG. Where are we with the use of N-acetylcysteine as a preventive and adjuvant treatment for COVID-19 ? Eur Rev Med Pharmacol Sci 2022;26:715–21.

[125]	Andreou A, Trantza S, Filippou D, Sipsas N, Tsiodras S. COVID-19 : The Potential Role of Copper and N-acetylcysteine ( NAC ) in a Combination of Candidate Antiviral Treatments Against SARS-CoV-2. In Vivo (Brooklyn) 2020;34:1567–88. https://doi.org/10.21873/invivo.11946.

[126]	Barlattani T, Celenza G, Cavatassi A, Minutillo F, Socci V, Pinci C, et al. Neuropsychiatric Manifestations of COVID-19 Disease and Post COVID Syndrome: The Role of N-acetylcysteine and Acetyl-L-carnitine. Curr Neuropharmacol 2025;23:686–704. https://doi.org/10.2174/011570159X343115241030094848.

[127]	Wrotek A, Badyda A, Jackowska T. Molecular Mechanisms of N-Acetylcysteine in RSV Infections and Air Pollution-Induced Alterations : A Scoping Review. Int J Mol Sci 2024;25:6051. https://doi.org/10.3390/ijms25116051.

[128]	Zoofaghari S, Forghani M, Dorooshi G, Maghami-mehr A. The role of N-acetyl cysteine and some other clinical antidotes in the treatment of patients with COVID-19 ; review of the current evidence. Immunopathol Persa 2023;9:e31406. https://doi.org/10.34172/ipp.2022.31406.

[129]	Mohanty RR, Padhy BM, Das S, Meher BR. Therapeutic potential of N-acetyl cysteine ( NAC ) in preventing cytokine storm in COVID-19 : review of current evidence. Eur Rev Med Pharmacol Sci 2021;25:2802–7.

[130]	Ejigu DA, Abay SM. N-Acetyl Cysteine as an Adjunct in the Treatment of Tuberculosis. Tuberc Res Treat 2020;2020. https://doi.org/10.1155/2020/5907839.

[131]	Mapamba DA, Sauli E, Mrema L, Lalashowi J, Magombola D, Buza J, et al. Impact of N -Acetyl Cysteine ( NAC ) on Tuberculosis ( TB ) Patients — A Systematic Review. Antioxidants 2022;11:2298. https://doi.org/10.3390/antiox11112298.

[132]	Schloss J, Leach M, Brown D, Hannan N, Kendall-reed P, Steel A. The effects of N -acetyl cysteine on acute viral respiratory infections in humans : A rapid review. Adv Integr Med 2020;7:232–9. https://doi.org/10.1016/j.aimed.2020.07.006.

[133]	Paraskevas T, Kantanis A, Karalis I, Michailides C, Karamouzos V, Koniari I, et al. N-acetylcysteine efficacy in patients hospitalized with COVID-19 pneumonia : a systematic review and meta-analysis. ROM J INTERN MED 2023;61:41–52. https://doi.org/10.2478/rjim-2023-0001.

[134]	Pang Q, Li G, Cao F, Liu H, Wei W, Jiao Y. Clinical efficacy of Chinese herbs for supplementing qi and activating blood circulation combined with N-acetylcysteine in the treatment of idiopathic pulmonary fibrosis : A systematic review and network meta-analysis. PLoS One 2022;17. https://doi.org/10.1371/journal.pone.0265006.

[135]	Rogliani P, Calzetta L, Cavalli F, Matera MG, Cazzola M. Pirfenidone, nintedanib and N-acetylcysteine for the treatment of idiopathic pulmonary fibrosis: A systematic review and meta-analysis Paola. Pulm Pharmacol Ther 2016. https://doi.org/10.1016/j.pupt.2016.07.009.

[136]	Sun T, Liu J, Zhao DW. Efficacy of N -Acetylcysteine in Idiopathic Pulmonary Fibrosis: A Systematic Review and Meta-Analysis. Medicine (Baltimore) 2016;95. https://doi.org/10.1097/MD.0000000000003629.

[137]	Zhang XL, Cao Y, Zheng B. Efficacy of N ‑ acetylcysteine plus pirfenidone in the treatment of idiopathic pulmonary fibrosis : a systematic review and meta ‑ analysis. BMC Pulm Med 2023;23:479. https://doi.org/10.1186/s12890-023-02778-w.

[138]	Huang C, Kuo S, Lin L, Yang Y. The efficacy of N -acetylcysteine in chronic obstructive pulmonary disease patients : a meta-analysis. Ther Adv Respir Dis 2023, 2023;17:1–12. https://doi.org/10.1177/17534666231158563.

[139]	Jiang C, Zou J, Lv Q, Yang Y. Systematic review and meta-analysis of the efficacy of N-acetylcysteine in the treatment of acute exacerbation of chronic obstructive pulmonary disease. Ann Palliat Med 2021;10:6564–76. https://doi.org/10.21037/apm-21-1138.

[140]	Prabu OG, Islami NN, Mellenia J, Nugroho P, Rajabto W, Shatri H. N-Acetylcysteine Role in Maintaining RenalFunction in Cancer Patients with Cisplatin-BasedChemotherapy. Int J Nephrol Renovasc Dis 2025;18. https://doi.org/10.2147/IJNRD.S563298.
